# Revealing low-temperature plasma efficacy through a dose-rate assessment by DNA damage detection combined with machine learning models

**DOI:** 10.1038/s41598-022-21783-3

**Published:** 2022-11-01

**Authors:** Amal Sebastian, Diana Spulber, Aliaksandra Lisouskaya, Sylwia Ptasinska

**Affiliations:** 1 Radiation Laboratory, University of Notre Dame, Notre Dame, IN 46556 USA; 2 Department of Physics and Astronomy, University of Notre Dame, Notre Dame, IN 46556 USA; 3 Department of Applied and Computational Mathematics and Statistics, University of Notre Dame, Notre Dame, IN 46556 USA

**Keywords:** Chemistry, Energy science and technology, Physics

## Abstract

Low-temperature plasmas have quickly emerged as alternative and unconventional types of radiation that offer great promise for various clinical modalities. As with other types of radiation, the therapeutic efficacy and safety of low-temperature plasmas are ubiquitous concerns, and assessing their dose rates is crucial in clinical settings. Unfortunately, assessing the dose rates by standard dosimetric techniques has been challenging. To overcome this difficulty, we proposed a dose-rate assessment framework that combined the predictive modeling of plasma-induced damage in DNA by machine learning with existing radiation dose-DNA damage correlations. Our results indicated that low-temperature plasmas have a remarkably high dose rate that can be tuned by various process parameters. This attribute is beneficial for inducing radiobiological effects in a more controllable manner.

## Introduction

Low-temperature plasmas (LTPs), generated from an electrical discharge at atmospheric pressure and room temperature, are reservoirs of diverse physical and chemical components that can be delivered to and significantly affect any biological target. The reactive ability and tunability of plasma properties have triggered a surge in scientific interest in basic and applied interdisciplinary research on LTPs. In their applications, LTPs have been integrated into modern strategies for medical treatments in which plasma can act as a unique source of radiation^1–3^. The potential of LTPs now extends to the inactivation of the SARS-CoV-2 virus^4–6^, in addition to its already proven clinical efficacy in cancer therapy^7–9^ and wound healing^10,11^.

One critical aspect that dictates the therapeutic efficacy and safety of irradiation is the dose rate, which is the radiation energy absorbed per unit of mass of the target within a unit of time. The rate spans an extensive range of values of grays per second (Gy/s) in medical settings^12^. Different radiation dose rates are deliberately used for different medical purposes because there is a strong correlation between the dose-rate level and the biological effects, such as cell survival, DNA damage, and gene expression^13^. In practice, the dose rate of the specific type of radiation is measured by detectors or dosimeters, or calculated, or both. Different types of dosimeters are used depending on several factors, such as the nature of the radiation, the dosimeter’s physical form, and the mechanisms employed to detect the dosimeter’s response to radiation. For example, chemical dosimeters, such as the Fricke dosimeter, are used as a primary standard of the dose absorbed in water and are valid to calibrate the absorbed dose in liquids, or the so-called water-equivalent dose. If potential health effects need to be assessed, a biodosimeter such as the alanine dosimeter is used because its composition is similar to living tissue. Thus, the so-called tissue-equivalent dose can be obtained with this type of dosimetry. A dosimeter is chosen according to its properties that evolve as a function of the dose rate delivered to the target exposed to ionizing radiation.

Traditional chemical dosimetry^13–17^ and biodosimeters^18^ have been employed to assess the radiation dose from LTPs. However, they were mostly limited to quantifying the specific species typically generated from water radiolysis (Fricke dosimetry) and persistent radicals derived from the amino acid (alanine dosimetry).

The properties of LTPs, and, therefore, the physical, chemical, and biological responses resulting from its radiation, can be combined and tuned by changing numerous plasma process parameters, such as the type of electrical discharge, pulse voltage characteristics, irradiation time, composition of the feed gas and its flow rate, and even ambient conditions, to name a few^19–23^. Another methodology used the equivalent total oxidation potential, which relies on all reactive oxygen and nitrogen species and UV emissions produced by LTP to determine the plasma dose^24^. Thus, in the context of LTP radiation, which results in various effects, there are limitations and uncertainties related to the usage of a specific dosimeter to measure the plasma dose rate.

Moreover, in contrast to other types of radiation, LTP has a complex composition of species, in which there is a synergistic interplay between plasma energetics and chemistry. This restricts the possible computational methods that can be used to calculate the plasma dose rate as conventionally defined in radiation research.

Therefore, new strategies for the routine determination of delivered plasma doses remain a challenge, and it is an emerging need to translate LTP applications from lab-based research into clinical treatment^3,9,25^.

In this work, we developed an innovative strategy by building a supervised machine learning (ML) framework that incorporated an extensive experimental database obtained in our laboratory, literature-based dose rates reported for different types of radiation, and specific deep learning algorithms for generating physically consistent predictive models. The framework consisted of a task workflow that aimed to predict the plasma dose rate from the extent of DNA damage, an indicator of the biological efficacy of LTP, which occurred at specific plasma parameters. The workflow involved finding the irradiation time that corresponded to the extent of DNA damage in the aqueous solution induced by LTP and then comparing it with those times obtained from the correlation between absorbed dose and aqueous DNA damage induced by other known types of radiation. Thus, the dose rate for LTP modeled at any combination of plasma process parameters would allow us to assess the ability of plasma to offer competent therapeutic efficacy with ensured radiation safety^14,26–29^. We also measured the LTP dose rates using two traditional dosimeters at a given set of process parameters and compared the values obtained with those computed using the framework.

## Results

### Generation of predictive models and LTP dose-rate extraction

First, we generated a robust predictive model that provided the extent of plasma-induced DNA damage for a given set of process parameters. The experimental data used to create the predictive model were acquired through the approach of design of experiments (DoE) using a helium-fed LTP source and the plasmid DNA as a biomolecular target^20,22^ (see Supplementary Information (SI) Fig. S1). For the DoE, we assessed the impact of four crucial LTP process parameters, such as the applied voltage, frequency, irradiation time, and feed gas flow rate, on plasma-induced DNA damage which we analyzed using electrophoresis (Fig. S2, Table S1). The data obtained and incorporated into the DoE matrix were split into training and testing data, and predictive modeling was implemented following the standard supervised ML workflow (Fig. S3). The data distribution for the DNA damage (Fig. S4) revealed many rare values, most of which corresponded to DNA damage at relatively short irradiation times. The rare values that correspond to the minority data were oversampled at the refining stage of the ML algorithm model (Fig. S5) using the synthetic minority oversampling algorithm^30^ (Fig. S6).

**Figure 1:**
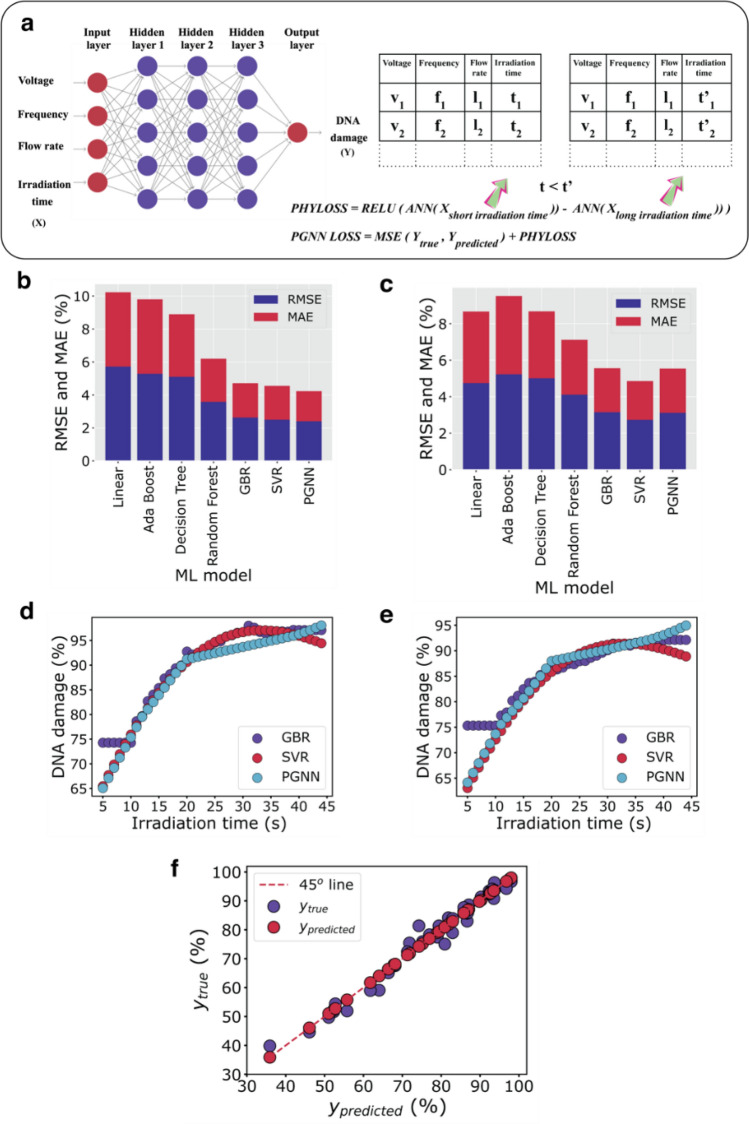
Overview of the predictive modeling. (**a**) Schematic diagram of the implementation of PGNN. Summary of evaluation scores on the test data (**b**) and cross-validation scores (**c**) for the different ML models. The metrics include root-mean-squared error (RMSE) and mean absolute error (MAE). Time dependence for DNA damage was modeled for two sets of process parameters: 10 kV, 4 kHz, and 2 standard liters per minute (slm) (**d**), and 11 kV, 3 kHz, and 2 slm (**e**), indicating physical violation for GBR and SVR. (**f**) Evaluation plot of the un-augmented test data for PGNN. Evaluation scores are R^2^ = 0.976, RMSE = 2.45, and MAE = 2.2.

Our experimental studies showed an increase in the extent of DNA damage with longer irradiation times for all combinations of other process parameters (Fig. S7). As expected, this occurred due to more interactions of plasma species, especially radicals with the target^9^. This physical phenomenon showed a linear dependence in a specific time regime, after which a slower accumulation of damage appeared. Therefore, to develop predictive models that obey this time-dependence correlation, we considered a physics-guided neural network (PGNN)^31^, which is a sophisticated deep learning algorithm based on an artificial neural network model (Fig. 1a). The implemented PGNN incorporated a physical loss function (Fig. 1a) that captured any violation in the data that showed any deviation from this time dependence. Then, we evaluated the test data set using the cross-validated model acquired from hyperparameter optimization (Fig. S5). The test data evaluation scores and cross-validation (CV) scores for PGNN, as well as those for other implemented ML algorithms, are summarized in Fig. 1b,c. Based on these scores, we identified three algorithms with the best performance for the predictive model: a gradient boosting regressor (GBR); support vector regression (SVR), and PGNN. However, our further inspection indicated that, although GBR and SVR offer impressive CV and evaluation performance, they fail to preserve the physical consistency for irradiation times shorter than 10 s and longer than 40 s, respectively (Fig. 1d,e, Fig. S8). In contrast, PGNN, in addition to providing the best scores (Fig. 1f), obeys the physical consistency for time dependence (Fig. 1d,e). Thus, PGNN was selected and used as a predictive model for the next task of the framework, which was to extract the dose rate for LTP radiation.

**Figure 2:**
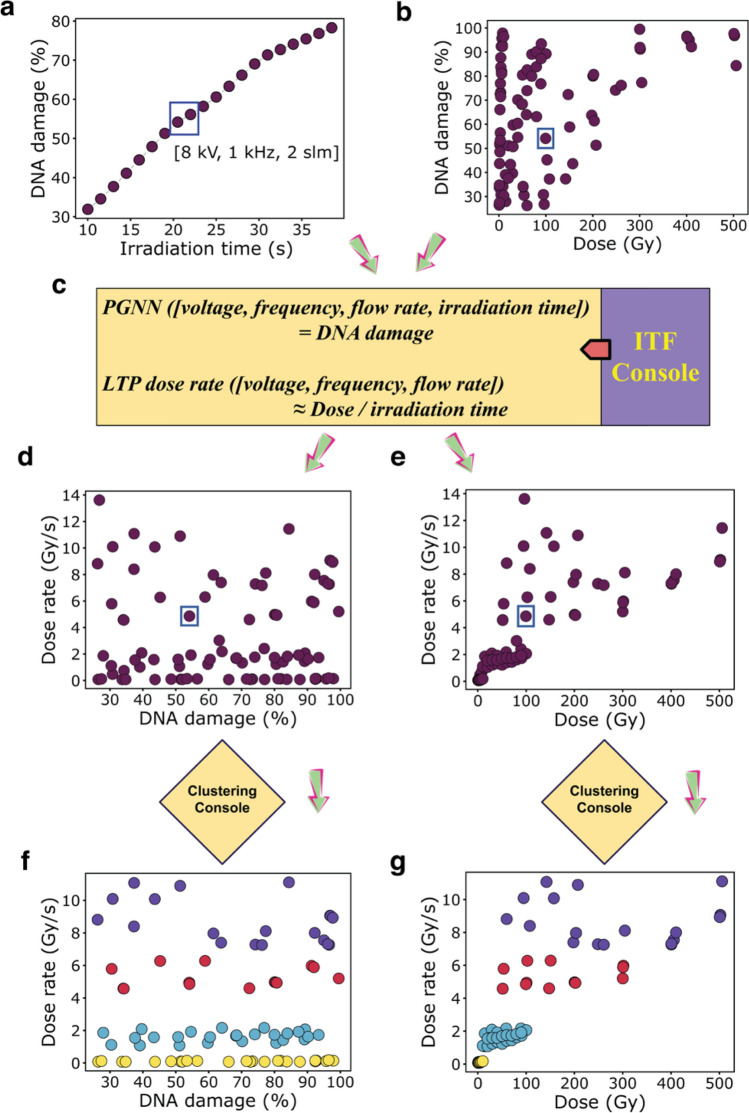
Workflow for the extraction of dose-rate values. (**a**) LTP-induced DNA damage versus irradiation time as modeled using PGNN for process parameters: 8 kV, 1 kHz, and 2 slm. (**b**) Dose-DNA damage correlations for gamma rays obtained from the literature. (**c**) Illustration of the ITF console used to obtain the dose-rate value for a selected data point, which is highlighted by the blue square in (**b**). Dose rates versus DNA damage obtained from the ITF console (**d**), and the transformed graph when clustering was performed (**f**). The clustering revealed four different clusters, represented by color: yellow, cyan, red, and purple (Table S2). Dose rate versus absorbed dose before (**e**) and after clustering (**g**).

Next, we created a LTP dose-rate extraction framework that combined the predictive model and the correlation between the absorbed dose and radiation-induced DNA damage (hereafter denoted as dose-DNA damage) for particulate and electromagnetic radiation, including electrons, ions, gamma rays, X-rays, and UV rays (Fig. S9). Based on the initial assumption that these dose-DNA damage correlations corresponded to the extent of DNA damage for LTP radiation, we collected the dose-DNA damage data from the rich body of literature reported over decades by several research groups (see Methods), and the data for plasma-induced DNA damage were systematically measured in our laboratory. In this task, we generated an irradiation time finder (ITF) console, which predicted the irradiation time in which LTP will cause the same extent of DNA damage as other types of radiation. For example, Fig. 2 shows the workflow that we implemented to find the dose rate for LTP from the data in the existing literature for dose-DNA damage for gamma rays (see Methods). These data were then fed into the ITF console, which operated by blending the predictive model with the dose database. Specifically, it provided the irradiation time (for LTP radiation, at a given set of three process parameters: voltage, frequency, and flow rate), which is the time at which plasma produced the equivalent extent of DNA damage (Fig. 2a) corresponding to the highlighted point in the dose-DNA damage database (Fig. 2b). Then, following our initial assumption of the equivalency of dose-DNA damage correlations for both types of radiation, the LTP dose rate was approximated as in Eq. (1):

1 $$LTP\, dose \,rate \left[voltage,\, frequency,\, flow\, rate\right]=\frac{dose}{{t}_{irr}},$$

where t_irr_ is the irradiation time estimated through the ITF console (Fig. 2c) for the highlighted point in the dose-DNA damage plot (Fig. 2b). We repeated this procedure for each data point in the dose-DNA damage database (Fig. 2b), which resulted in generating LTP dose rates corresponding to the equivalent extent of DNA damage and the absorbed dose as seen in Fig. 2d,e. Both plots revealed that the estimated dose-rate values are spread over a wide range from 0.1 to 15 Gy/s. Therefore, we needed to cluster the dose-rate values into four distinct data groups (Fig. 2f,g). We accomplished this by incorporating a clustering console to convert the dose rates into clusters through K-means clustering of the dose-rate values followed by outlier treatment for each cluster. We then computed the centroid (i.e., the mean of each cluster) and standard deviation in dose rates for each cluster (Table S2). Similarly, we encountered spread patterns in dose-rate plots for other types of radiation (Fig. S10), and we followed the clustering procedure to obtain the values for all clusters, as summarized in Table S3. It is important to note that formation of clusters in a wide range of dose rates can be attributed to different types of DNA and buffers used, as well as other experimental factors in the data reported in the literature which were incorporated into our modeling.

### LTP dose-rate dependence on plasma process parameters

Our LTP dose-rate framework evaluated the dose rate for LTP at a given set of process parameters; however, plasma-induced DNA damage evolves when these parameters are varied. Therefore, we investigated the evolution of dose rates for different process parameters in our experiments. For example, Fig. S11 shows the effect of voltage and frequency on the dose-rate values at a constant flow rate of 2 standard liter per minute (slm) for LTP, at which the extent of DNA damage at the specific irradiation time is equivalent to the damage by gamma radiation. With an increase in frequency, a centroid of dose-rate values increases up to as much as 20 Gy/s for this case (Fig. S11b,c, Movie S1). Although an increase in the dose-rate with voltage also occurs, the effect is less significant than the one observed with a rise in frequency (Fig. S11d,e, Movie S2).

**Figure 3:**
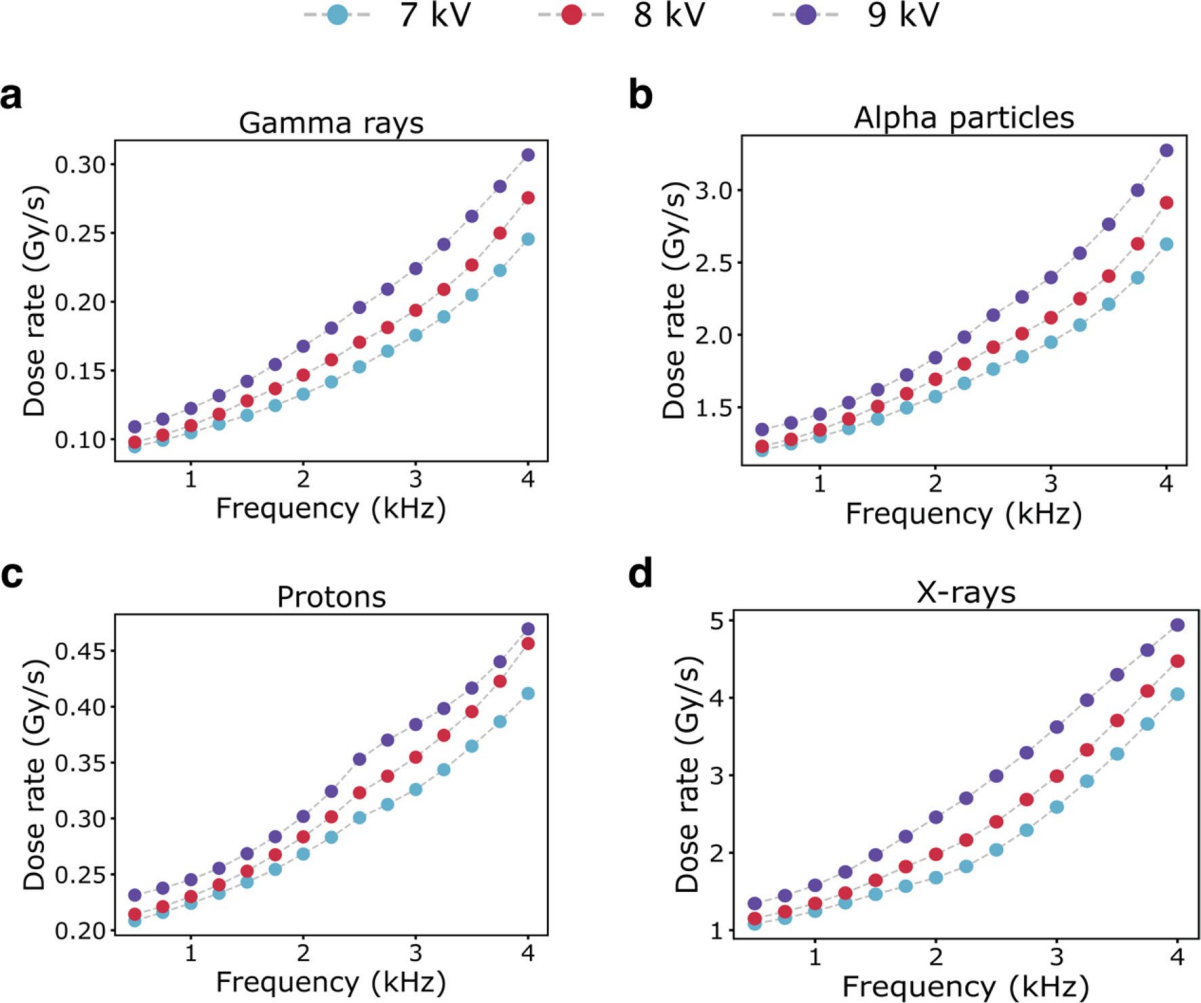
Dose-rate dependence with frequency and voltage. The plot of average dose rate (centroid) versus frequency for the lowest dose-rate cluster as modeled using dose-DNA damage correlation for gamma rays (**a**), alpha particles (**b**), protons (**c**), and X-rays (**d**) at three different applied voltages (7 kV, 8 kV, and 9 kV). The frequency was varied from 0.5 to 4 kHz at increments of 0.25 kHz.

Similarly, we computed the evolution of the dose-rate dependence with voltage and frequency if the DNA damage by LTP is equivalent to the effects from other radiation types. Figure 3 and Fig. S12 represent the average dose rate estimated for clusters with the lowest dose rate for LTP compared with different radiation types. Generally, in radiation research, the dose-rate effect is associated with the yield of specific reactive species, which induce chemical alterations in the biomolecular target^13^. Using a traditional chemical dosimeter in our previous study, we observed an increase in the total amount of reactive species as a function of frequency and voltage for the same LTP source^15^. Thus, the increase in the estimated dose rate with a frequency and voltage in this study can be related to the increase in the number and/or type of plasma species responsible for DNA damage. However, our ML framework has not yet included calculations on any possible chemical reactions upon LTP irradiation. Nevertheless, the prospect of adjusting the dose rate of LTP by tuning the process parameters offers great potential for a wide range of applications in medical treatments^32,33^.

### Comparison of modeled and measured LTP dose-rate values

**Figure 4:**
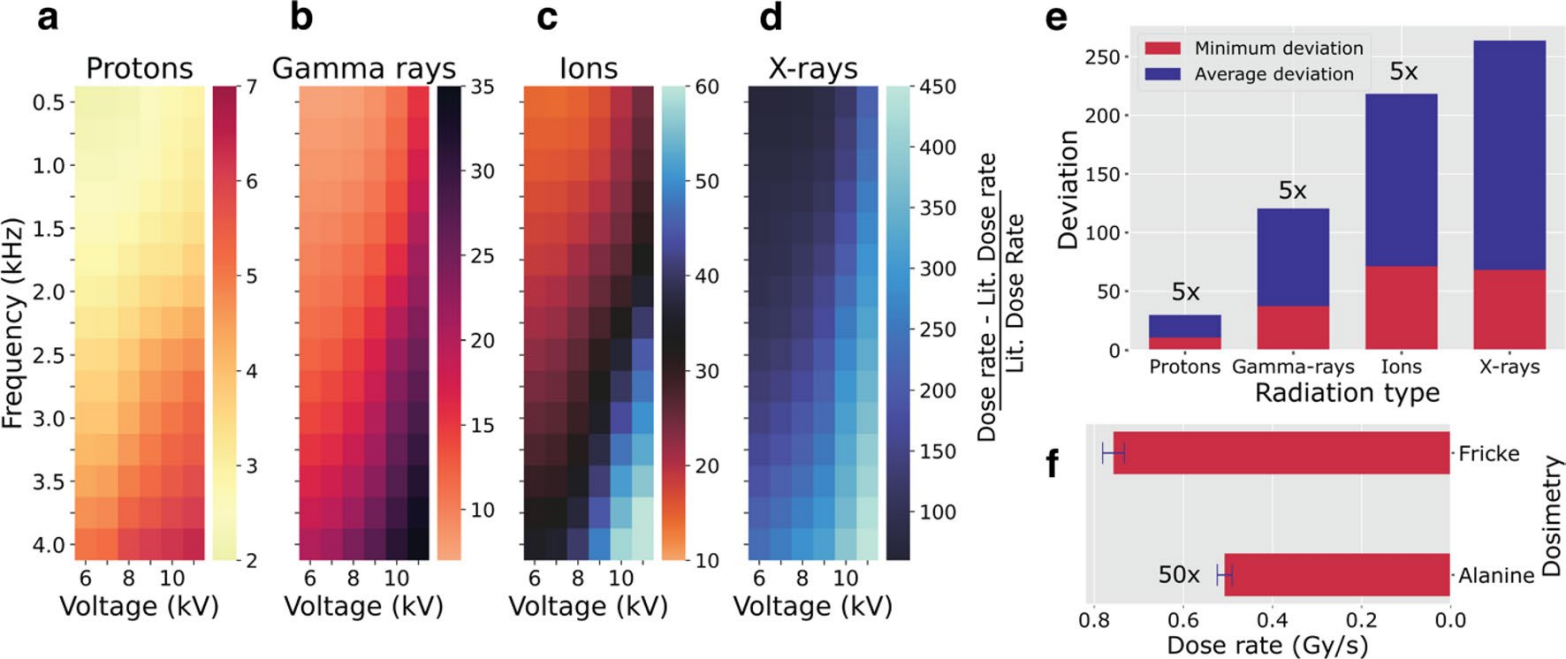
Comparison of LTP dose rates with dose rates for other types of radiation. Heat maps of the relative deviation of the estimated dose rate of LTP with the literature values for protons (**a**), gamma rays (**b**), ions (**c**), and X-rays (**d**) for the lowest dose-rate cluster. (**e**) Summary of the minimum relative deviation of dose rate and average relative deviation of dose rate for different radiation types. See the SI about excluding UV rays, electrons, and alpha particles in our dose-rate comparison. (**f**) The experimental results of LTP dose-rate measurements using the Fricke and alanine dosimetries irradiated at the following process parameters, 8 kV, 1 kHz, and 2 slm. See the “Methods” section and Figs. S13 and S14 for the detailed methodology and data analysis.

The above described LTP dose-rate extraction framework was based on the initial assumption that the effects of LTP on DNA damage would correspond with the one from the dose-DNA damage correlation by finding the irradiation time for a given radiation type. Therefore, this assumption could possibly insinuate that the estimated value of the dose rate for LTP will be the same as the measured dose rate for the type of radiation used for finding this value. However, as will be further shown, that is not the case. In addition, the LTP dose rate evolves with different process parameters (Fig. 3). Thus, to determine how the estimated dose rate for LTP relates to other radiation types, we introduced a comparative task into the ML framework. We created metrics of the estimated doses and evaluated their deviation from the dose-rate values found in the literature for a given radiation type. Heat maps in Fig. 4a–d represent the obtained relative deviation with dose-rate values reported for four types of radiation for the cluster with lowest dose-rate value. The heat maps for different process parameters show the maximum disparity at a high voltage (> 9 kV) and frequency (> 2 kHz), because these parameter combinations generated high estimated dose rates for LTP (Fig. 3). Table S4 summarizes the other metric values used for dose-rate comparison, including the ratio of the minimum estimated dose rate of LTP for the cluster with the lowest dose rate and the dose rate found in the literature for a given radiation type reported for DNA irradiation. The bar chart in Fig. 4e represents the average and minimum relative deviations of the LTP dose rate and the literature values, indicating the slightest deviation between LTP and the proton’s dose rates and the maximum disparity with X-rays. The closest match between the minimum LTP dose rate estimated through our modeling is with the dose rate for protons. This minimum dose rate for LTP has a magnitude of 0.2 Gy/s, which is remarkably high compared to other types of radiation.

We measured the LTP dose rates with two dosimeters, the Fricke and alanine dosimeters (Fig. 4f). We selected these two methods because the former is used typically to estimate dose rates of liquids and yields of water products and the latter for biological efficacy; thus, they were relevant to the type and form of our target, an aqueous DNA.

Both dosimeters were irradiated by LTP at the same plasma conditions, which corresponded to the process parameters for which the dose-rate values were estimated using the ML framework (Tables S2, S3). A Fricke (ferrous-ferric ion) chemical dosimeter is widely used in radiation chemistry for the overall detection of water radiolysis products such as the hydroxyl radical, hydrogen superoxide/hydrogen radical, and hydrogen peroxide^34^. In this method, during ionizing radiation, a ferrous ion (Fe^2+^) is oxidized to a ferric ion (Fe^3+^), which has optical absorption at ~ 304 nm; therefore, the absorbed dose can be determined based on the concentration of Fe^3+^^35,36^. This method indicated a substantially high dose rate of approximately 0.76 Gy/s, four times higher than the minimum dose rate for LTP of 0.2 Gy/s as stated above, obtained from the modeling for the same plasma process parameters.

Alanine dosimetry is based on radical detection using an electron paramagnetic resonance (EPR) spectroscopy^37^ that can be used over a wide dose range of 1 Gy to 150 kGy for all radiation types^38^. The dose rate obtained using alanine was 0.01 Gy/s, nearly two orders of magnitude lower than the dose rate obtained by Fricke dosimetry. The difference indicated that the two traditional dosimetry methods for assessing LTP dose rates gave inconsistent results and that alternative methods were therefore needed.

## Discussion

LTP is an emerging type of radiation that has shown already significant potential in various therapeutic applications. It could soon be used in clinical practice if the plasma radiation dose could be established. The commonly used dosimetric procedures in radiation research, which have been utilized for decades for high-energy ionizing radiation, have proven to be insufficient for providing accurate measurements of the LTP dose rate, which is a key quantity of any radiation source in clinical usage. Strategies have been proposed to overcome this challenge by defining the plasma dose in new ways^3^. We showed that using two different dosimetric methodologies resulted in two distinctive dose-rate values and this was caused by several factors. The primary factors were different mechanisms of radiation responses due to plasma irradiation and the fact that the plasma species’ penetration and diffusivity depend on the physical form of dosimeters. For example, we measured a lower absorbed dose rate for the alanine dosimeter, which is a solid target, compared to the Fricke dosimeter, which is a liquid target, that was most likely attributed to lower penetration depths in solids compared to liquids. In addition, the dose rates were slightly, but noticeably, different when the Fricke solution was stirred or unstirred during irradiation. Also, the ionic strength of the Fricke solution and the aeration conditions^39^ could affect the reaction rates for the formation of water radiolytic products. With alanine dosimetry, we observed better reproducibility of the results; however, the solid form of this dosimeter limited the diffusion and penetration of plasma species, causing EPR signal saturation relatively quickly.

Therefore, in this work, we proposed an alternative method that could improve our determination of the LTP dose rates by using aqueous DNA and the damage to it; this would be an indicator of radiobiological efficacy for a dosimeter that combines the characteristics of water- and tissue-dose equivalency. To elucidate this, we generated a dose-rate assessment ML framework that incorporated a predictive model of plasma-induced DNA damage based on our experimental data with the correlations of dose-DNA damage identified in the existing literature. In this framework, we implied the equivalency of the extent of DNA damage induced by LTP with other types of radiation having a known absorbed dose. Then we obtained the LTP dose rates by finding the irradiation times that corresponded to this equivalency. The predictive model, which obeyed physical consistency, unraveled the dose-rate evolution of LTP over a wide range of two process parameters, applied voltage and frequency. Varying dose rates of plasma are of potential interest in therapeutic applications in which specific biological effects can be targeted. The strategy in which deep learning algorithms were incorporated for generating predictive control models was recently utilized in a prototypical setup for the delivery of a safe dose of plasma^40^.

Furthermore, we extended our methodology to show the effectiveness of LTP for DNA damage in relation to that of different radiation types by comparing the estimated plasma dose rate with the rates reported in the literature. We also compared the modeled values with those measured by two common dosimetric techniques.

Our experimental studies and ML framework did not include the effects of other factors (e.g., the type of DNA and buffer used) or other plasma process parameters (e.g., the duty cycle of the plasma pulse) on DNA damage. Thus, our framework excluded any deviations that could arise because of a change in these parameters. More investigations should be performed in future to assess these parameters’ effect on the dose-rate estimation.

Nevertheless, our conclusion that LTP can provide high dose rates (0.2 Gy/s), even higher than those of protons, is a promising and striking outcome. It revealed a value between those obtained from two dosimetric methods.

Despite the high dose-rate effect of LTP, it is considered a safe radiation source for treatment^14,26–29^. Therefore, LTP is a beneficial therapeutic tool for diverse clinical applications, particularly for situations in which high dose-rate radiation is required to induce specific biological outcomes.

## Methods

### Plasma-induced DNA damage and dosimetry: experiment

The helium-fed, LTP source used for our experiments was operated based on a dielectric barrier discharge, and it had the same design (Fig. S1) as the one implemented in our previous studies^41^. We used pUC18 plasmid DNA (Thermo Fisher Scientific, Waltham, MA) as a target for LTP irradiation. Plasmid DNA was placed under the tube orifice for LTP irradiation at several different sets of process parameters. After irradiation, we collected DNA and loaded it to the agarose gel for further processing electrophoresis technique (Biorad Inc, Hercules, CA) and imaging methods to quantify the extent of plasma-induced DNA damage, that is the percentage of strand breaks in the DNA and its denaturation. We followed the methodology described in the SI.

Finally, we incorporated the data into the design of the experiments matrix for the generation of the dataset, which we used for predictive modeling.

We used a Fricke dosimeter that contains 1.4 mM Fe^2+^ (iron (II) sulfate heptahydrate from Sigma Aldrich Inc, St. Louis, MO) in 0.4 M H_2_SO_4_ saturated with O_2_. We used alanine pellets (GEX corporation, Centennial, CO) having a 4 mm diameter and 2.35 mm thickness, and a composition of 93% of pure l-ɑ-alanine.

We carried out EPR measurements using Bruker EMXplus spectrometer (Bruker Corporation, Billerica, MA) with ER4119HS standard resonator in X-band (9.77 GHz). Next, we determined the response of alanine dosimeters to plasma irradiation by comparing them to those after ^60^Co gamma-ray irradiation.

### Generation of dose-DNA damage database

Our proposed dose-rate assessment framework for extracting dose rates for LTP radiation at various combinations of process parameters used the correlations from the literature reported between the absorbed dose and radiation-induced DNA damage (denoted as dose-DNA damage) for different types of radiation (Fig. S9). The first step for executing the proposed strategy was to create the database of dose-DNA damage correlation by performing a literature survey. The extensive literature survey consisted of dose-DNA damage correlations for the following types of radiation: alpha particles, gamma rays, X-rays, ions such as carbon, iron, and helium, protons, electrons, and UV rays. Based on the literature data, we generated the dose-DNA damage correlations for all types of radiation (alpha particles, gamma rays, ions, protons, UV rays, electrons and X-rays) that were implemented into the workflow of LTP dose-rate extraction.

### Supervised machine learning (ML) models

We used the standard supervised ML workflow to perform predictive modeling of the total plasma-induced damage to DNA damage (Fig. S3). Initially, the overall data acquired from DoE were split into training and test data folds (75:25 ratio). Then, the training data were entered into an ML algorithm, and the refinement/learning using cross-validation was performed on the training data. The ML algorithms chosen for modeling included linear regression, decision tree regression, ensemble-based algorithms (random forest regression, gradient boosting regression, AdaBoost regression), support vector regression, etc.

The oversampling of the data was done during the model refinement stage, in which the minority data region (Fig. S4) in each training fold was augmented by the synthetic minority oversampling technique (SMOTE). The potential class imbalances that could occur during the augmentation of the minority regions were counterbalanced by sufficiently oversampling the majority region of the data. Model refining using hyperparameter tuning of the ML algorithm was performed in the learning phase and was implemented via grid-search cross-validation (CV). A fivefold grid-search CV was performed on the training data with SMOTE applied individually after the training and validation fold split (Fig. S5). We recorded the best CV scores for different ML algorithms and conducted the final assessment of the model by evaluating the refined cross-validated model on the unaugmented test data (Fig. S3).

One of the ML algorithms that we used to model plasma-induced DNA damage was a PGNN^31^. The extent of DNA damage increased as the irradiation time was extended (Fig. S7), causing a larger number of plasma interactions with DNA that provides one of the physical effects observed in our experiments. Therefore, we included the time dependence of DNA damage in our predictive modeling using PGNN. Any physical inconsistency could be captured because PGNN functions incorporated an extra loss term, namely the physical loss function (PHYLOSS), into the pure ANN loss function. In other words, any violation to the time dependence of total plasma-induced DNA damage can be eliminated in the modeling. Next, we used similar procedures to refine the model and for grid-search cross-validation, as we did for other ML algorithms; the only difference was that the extra physical loss function was incorporated to produce physically consistent predictions. We found that a three hidden-layer model architecture with 50 neurons provided supreme prediction performance and physical consistency from the hyperparameter tuning process using grid-search CV. More details on modeling are presented in the SI.

## Supplementary Information

Supplementary Information.

Supplementary Movie S1.

Supplementary Movie S2.

## Data Availability

The datasets used and/or analyzed during the current study are available from the corresponding author on reasonable request.

## Code availability

Python codes that were used in the analysis are available on request from the authors (S. Ptasinska and A. Sebastian).
