# Supplementary material for: Revealing low-temperature plasma efficacy through a dose-rate assessment by DNA damage detection combined with machine learning models
Source: Sci Rep. 2022 Nov 1;12:18353. doi: 10.1038/s41598-022-21783-3 (PMC9626482; doi:10.1038/s41598-022-21783-3)
Supplement: Supplementary file 1 — Supplementary Information. [file 41598_2022_21783_MOESM1_ESM.pdf]

Supplementary Information for

Revealing low-temperature plasma efficacy through a dose-rate assessment by DNA damage detection combined with machine learning models

Amal Sebastian[1,2], Diana Spulber[1,2,3], Aliaksandra Lisouskaya[1], and Sylwia Ptasinska[1,2*]

[1]Radiation Laboratory, University of Notre Dame, Notre Dame, Indiana 46556, USA
[2]Department of Physics and Astronomy, University of Notre Dame, Notre Dame, Indiana 46556, USA
[3]Department of Applied and Computational Mathematics and Statistics, University of Notre Dame, Notre Dame, Indiana 46556, USA
[*]sptasins@nd.edu

This PDF file includes:

Supplementary text
Figures S1 to S14
Tables S1 to S4
Legends for Movies S1 to S2
SI References

Other supplementary materials for this manuscript include the following:

Movies S1 to S2

Supplementary Information Text

An extended technical description of experimental and computational methods

Low-temperature plasma source

The helium-fed, LTP source used in this work was operated based on a dielectric barrier discharge, and it had the same design (Fig. S1) as the one implemented in our previous investigations[1]. Briefly, the LTP source consisted of a pair of tubular brass electrodes (50 mm in length) separated by a distance of 30 mm along a fused silica tube. The tube had an outer diameter of 6 mm and an inner diameter of 5 mm. Ultra-high pure research-grade helium of, 99.999%, (Airgas Inc., Radnor, PA) flowed through the tube. One of the brass electrodes was grounded, and high voltages that ranged from 8 to 11 kV were applied to the other electrode through a custom-built, high-voltage, direct current power supply, leading to the ignition of the plasma discharge in the tube. Fig. S1 shows a typical current-voltage discharge characteristic. Then, the plasma was launched through a tube orifice in the air, forming a jet that was in contact with a target during irradiation. Thermalization during plasma ignition was prevented by pulsing the applied voltage through a pulse generator (80 MHz Function/Arbitrary Waveform Generator, 33 250 A, Agilent Tech., Santa Clara, CA). The frequency of the pulse ranged from 1 kHz with a pulse width of 500 µs to 4 kHz with a pulse width of 125 µs, keeping a constant duty cycle of 50%. We used a mass flow controller (MASS-VIEW flow regulator, MV-394-He, Bronkhorst High-tech., Bethlehem, PA) to regulate the flow rate of the feed gas passing through the tube in the range of 2 to 4 standard liters per minute (slm). In this work, we varied four LTP process parameters, i.e., the applied voltage, frequency, irradiation time, and the flow rate of the feed gas.

Plasmid DNA

We used pUC18 plasmid DNA (Thermo Fisher Scientific, Waltham, MA) as a target for LTP irradiation. Plasmid DNA that was extracted from E. coli bacteria and that contained 2686 base pairs was diluted with deionized water to obtain 100 ng of DNA in a 15 µl solution in each sample. Then, we injected the DNA solution into a glass well (Fig. S1) and placed it under the tube orifice for LTP irradiation. After irradiation, we collected DNA from the glass well by pipetting from each well and loaded it to the agarose gel for further processing (see the next section).

Agarose gel electrophoresis

We used agarose gel electrophoresis (Biorad Inc, Hercules, CA) and imaging methods to quantify the extent of plasma-induced DNA damage, that is the percentage of strand breaks in the DNA and its denaturation. We followed the exact methodology used in our previous studies[2,3]. Briefly, the methodology involved loading the plasma-irradiated DNA in the 0.8% agarose gel stained with SYBR green (Bioscience Lonza, Morrisville, NC). Next, the prestained gel was electrophoresed with a running time of 3 hours at a voltage of 70 V (Gel set, Biorad Inc, Hercules, CA). We visualized the electrophoresed gel with a UV fluorescence imager (Molecular imager Gel Doc XR, Biorad Inc, Hercules, CA). Typically, a gel showed bands of DNA at four positions with different intensities (Fig. S2). The band positions corresponded to single-strand breaks (SSBs) and double-strand breaks (DSBs) in DNA, undamaged supercoiled DNA (SC), and DNA denaturation (D). The fluorescence intensities of the DNA bands corresponded to the amounts of DNA and were quantified using software (Quantity one, Biorad Inc, Hercules, CA).

We obtained a percentage of total DNA damage, including SSBs, DSBs, and the denaturation of DNA in the treated sample from the UV-imaged gels, and then performed the following calculation (Eq. S1):

$$DNA\ damage\ (\%) = \frac{(I_{SSB}+I_{DSB}+I_D)}{(I_{SSB}+I_{DSB}+I_D+I_{SC})} \times 100 \qquad \text{(S1)}$$

where I_{SSB}, I_{DSB}, and I_D correspond to the fluorescence intensities of the DNA bands with a particular form of damage, and I_{SC} corresponds to undamaged DNA. Next, the extent of plasma-induced DNA damage was obtained by subtracting the fraction of total DNA damage in the non-irradiated control sample, i.e., the sample placed under gas flow without LTP ignition, from the total DNA damage in the sample irradiated by LTP with a given set of process parameters (see Fig. S2). Then, the calculated extent of DNA damage was averaged over multiple experimental trials (three to four) for the same plasma conditions. We repeated the procedure for samples at each combination of process parameters. Finally, we incorporated the data into the design of the experiments matrix for the generation of the dataset, which we used for predictive modeling.

Data acquisition by the design of experiments (DoE)

We performed data acquisition for the total plasma-induced DNA damage by implementing a statistical experimental design, also known as the design of experiments (DoE). The DoE involved assessing the impact of four crucial LTP process parameters on plasma-induced changes in the DNA sample. These parameters included the applied voltage, frequency, feed gas flow rate, and plasma irradiation time of DNA. We generated a DoE matrix at various combinations of the parameters with the values listed in Table S1. We performed all plasma irradiations at a distance of 2 cm between the DNA sample and the tube orifice.

We chose this distance because the length of the plasma jet was approximately 2-3 cm; thus, a 2-cm distance ensured that a substantial contact occurred between the plasma jet and DNA at each combination of the LTP process parameters.

Supervised machine learning (ML) models

We used the standard supervised machine learning (ML) workflow to perform predictive modeling of the total plasma-induced damage to DNA damage (Fig. S3). Initially, the overall data acquired from DoE were split into training and test data folds (75:25 ratio). Then, the training data were entered into an ML algorithm, and the refinement/learning using cross-validation was performed on the training data. The ML algorithms chosen for modeling included linear regression, decision tree regression, ensemble-based algorithms (random forest regression, gradient boosting regression, AdaBoost regression), support vector regression, etc.

The oversampling of the data was done during the model refinement stage, in which the minority data region (Fig. S4) in each training fold was augmented by the synthetic minority oversampling technique (SMOTE) (see the next section). The potential class imbalances that could occur during the augmentation of the minority regions were counterbalanced by sufficiently oversampling the majority region of the data. Model refining using hyperparameter tuning of the ML algorithm was performed in the learning phase and was implemented via grid-search cross-validation (CV). A fivefold grid-search CV was performed on the training data with SMOTE applied individually after the training and validation fold split (Fig. S5). We recorded the best CV scores for different ML algorithms and conducted the final assessment of the model by evaluating the refined cross-validated model on the unaugmented test data (Fig. S3).

Synthetic minority oversampling technique (SMOTE)

The distribution of the extent of plasma-induced DNA damage showed rare values (Fig. S4), corresponding primarily to the damage that occurred at short irradiation times (i.e., 10 s). The distribution indicated that most data for DNA damage were distributed over a value of 70%. Thus, a greater extent of plasma-induced DNA damage occurred at many combinations of process parameters. The rare data instances that corresponded to the minority data region were oversampled to address this class imbalance problem, and we performed the oversampling only during the model refinement stage in the predictive modeling. Oversampling was achieved through the SMOTE (Fig. S6), which is a common augmentation technique used to overcome the class imbalance in predictive modeling problems.

Physics-guided neural networks (PGNN)

One of the ML algorithms that we used to model plasma-induced DNA damage was a physics-guided neural network (PGNN)[4]. PGNN is a novel and sophisticated algorithm based on a pure artificial neural network (ANN) architecture, which has been used in recent years to generate models that were physically consistent[5-7].

The extent of DNA damage increased as the irradiation time was extended (Fig. S7), causing a larger number of plasma interactions with DNA that provides one of the physical effects observed in our experiments. Therefore, we included the time dependence of DNA damage in our predictive modeling using PGNN. Any physical inconsistency could be captured because PGNN functions incorporated an extra loss term, namely the physical loss function (PHYLOSS), into the pure ANN loss function. In other words, any violation to the time dependence of total plasma-induced DNA damage can be eliminated in the modeling. The PHYLOSS function was computed by considering two unlabeled datasets: X with a short irradiation time (t) and X' another with a longer irradiation time (t') (Fig. 1a) with the time ranging from 10 to 50 s. Each row in X and X' had the same values for voltage (v), frequency (f), and flow rate (l); only the irradiation time differed, which was longer for X' than for X for each row.

A loss function (PHYLOSS) that corresponded to the physical violations of the predictions at longer irradiation times (if higher than the short irradiation time predictions) was implemented by using the step rectifier linear unit (RELU) function, which has the general form shown in Eq. S2:

$$RELU(x) = 0, if\ x \leq 0\ and\ RELU(x) = x, if\ x > 0 \qquad (S2)$$

Hence, a meaningful and simple physical loss function with RELU was implemented via Eq. S3:

$$PHYLOSS = RELU[ANN(X) - ANN(X')] \qquad (S3)$$

In Eq. S3, ANN (X) corresponds to the ANN predictions for X, where X is the process parameter vector (i.e., voltage, frequency, flow rate, irradiation time).

The total loss before the weight update process is given by Eq. S4:

$$LOSS = MSE(ANN\ LOSS) + PHYLOSS \quad (S4)$$

In Eq. S4, MSE is the mean-squared-error between the predicted and true values, which is the standard loss function for a pure ANN regressor.

The extra physical loss function ensured that the weight update process in ANN learning also minimized the PHYLOSS, ensuring that a physically consistent model was generated during the model learning.

Next, we used similar procedures to refine the model and for grid-search cross-validation, as we did for other ML algorithms; the only difference was that the extra physical loss function was incorporated to produce physically consistent predictions. We found that a three hidden-layer model architecture with 50 neurons provided supreme prediction performance and physical consistency from the hyperparameter tuning process using grid-search CV.

Generation of dose-DNA damage database

Our proposed dose-rate assessment framework for extracting dose rates for LTP radiation at various combinations of process parameters used the correlations from the literature reported between the absorbed dose and radiation-induced DNA damage (denoted as dose-DNA damage) for different types of radiation (Fig. S9). The first step for executing the proposed strategy was to create the database of dose-DNA damage correlation by performing a literature survey[8-39].

Due to the limited data on the specific type of plasmid DNA that we used as a target for LTP irradiation (i.e., pUC18), we collected the dose-DNA damage data for several types of plasmid DNA (pBR322, pEC, pGEM-3Zf, etc.), thereby generating a rich body of data for our modeling. Therefore, our methodology neglected any deviations that could be caused by a type of plasmid DNA used for irradiation as well as a type of buffer solution. Most reported DNA studies used phosphate-buffered saline or Tris-based buffers.

The extensive literature survey consisted of dose-DNA damage correlations for the following types of radiation: alpha particles[8-11], gamma rays[8],[11-23], X-rays[24-29], ions such as carbon, iron, and helium[12,30-33], protons[17,34,35], electrons[9,36,37], and UV rays[38,39].

In most studies, the value of the absorbed dose was provided or plotted in figures; therefore, in these cases, we extracted the values from plots using the graph data extractor tool (Web Plot Digitizer, Version 4.5, open-source).

Based on the literature data we collected, we generated the dose-DNA damage correlations for all types of radiation (alpha particles, gamma rays, ions, protons, UV rays, electrons and X-rays) that were implemented into the workflow of LTP dose-rate extraction.

K-means clustering of dose rates

The dose rates estimated for LTP using our dose-rate assessment framework (Fig. 2) had a wide range of values for all types of radiation used to extract these values (Fig. S10). For example, the dose rate for LTP that was obtained using the dose-DNA correlation and the comparison of plasma-DNA damage to the extent of DNA damage induced by gamma rays showed values that ranged from 0.1 to ~15 Gy/s (Fig. 2c). Similarly, patterns with spread dose-rate values that correlated to the absorbed dose and the extent of DNA damage were observed for all other types of radiation (Fig. S10); therefore, we performed data clustering using a clustering console (Figs. 2d and 2e). For clustering, we used a rudimentary 1-dimensional K-means clustering. Briefly, K means clustering involved the computation of the distance of each dose-rate data instance with the centroids (mean) of different clusters, followed by assigning that data instance to the cluster with a minimum estimated distance. Initial values were provided for the centroids, and the centroid values were updated with the mean of the clusters obtained after the complete data assignment.

While performing clustering, we ensured that each cluster maintained an equivalent or a comparable number of data instances with the other clusters, which is crucial for preventing overfitting of the data. As a result, the clustering process yielded 2 to 4 clusters (Tables S2 and S3) for most types of radiation. Moreover, we calculated the dose rates for each computed cluster as their values varied with changes in the process parameters. We also investigated the evolution of clusters and the average dose rate (centroid of the cluster) when changing process parameters, such as the applied voltage and the frequency (Fig. 3 and Figs. S11 and S12).

Outlier treatment

We applied specific outlier removal and treatment methods to minimize the standard deviation of dose-rate values obtained for each cluster.

We used the two-sigma method for removing the outlier values for dose rates. We did this by disregarding the dose rates that had a dose-rate value less than the mean (dose rate) -2σ or higher than the mean (dose rate) + 2σ, where σ is the standard deviation of the dose-rate cluster. The updated cluster showed a minimum deviation in the dose rate.

To further minimize the spread of values of dose rates for some clusters, we used a percentile capping of dose-rate values. In this method, 95th percentile and 5th percentile capping were performed; we kept the value above the 95th percentile at the 95th percentile value and the values below the 5th at the 5th percentile value. The treatment was found to decrease the standard deviation of the dose-rate clusters.

Summary of deviation metrics used in the comparative task

Our dose-rate assessment framework after extracting dose rates for LTP radiation also involved creating suitable metrics that captured the deviation of the LTP dose rate with the dose-rate value for a specific type of radiation. The primary metric used in our comparison included the relative deviation metric parameter defined for the dose-rate cluster using Eq. S5:

$$Deviation = \frac{mean(LTP\ dose\ rate\ [voltage, frequency]) - mean\ (Literature\ value)}{mean(literature\ value)} \quad \text{(S5)}$$

where mean (LTP dose rate) corresponds to the average of the LTP dose-rate value for the cluster and mean (Literature value) corresponds to the average of the reported dose-rate values in the literature.

In addition to the relative deviation metric, we also used statistical metrics such as root-mean-squared error (RMSE) and mean absolute error (MAE), defined for the dose-rate cluster as shown in Eqs. S6 and S7.

$$RMSE = \sqrt{mean\ [(LTP\ dose\ rate\ [voltage, frequency] - Literature\ value)^2]} \quad \text{(S6)}$$

$$MAE = mean\ (||\ (LTP\ dose\ rate\ [voltage, frequency] - Literature\ value)||) \quad \text{(S7)}$$

Because RMSE and MAE can depend on the magnitude of the dose rates reported in the literature, they were scaled to the mean of the literature dose-rate values (Table S4).

As previously mentioned, the estimated dose rate for LTP varied depending on the process parameters. Therefore, we evaluated the metrics at a range of voltages from 6 to 11 kV, with 0.5 kV steps, and frequencies from 0.5 to 4 kHz, with 0.25 kHz steps, for a flow rate of 2 slm. We then computed the average of the deviation metrics, which are summarized in Table S4.

In addition, we estimated another important metric, the ratio of the minimum estimated value of the dose rate for LTP to the literature value of the dose rate for a particular type of radiation defined by Eq. S8:

$$Minimum\ deviation = \frac{minimum\ (mean\ (LTP\ dose\ rate))}{mean\ (Literature\ value)} \quad \text{(S8)}$$

Comparison of specific types of radiation with the estimated values of the LTP dose rate

In order to obtain the dose-rate values for LTP, we used the dose-DNA correlations for different types of radiation reported in the literature. In the following task, our aim was to compare the LTP dose-rate values obtained from our modelling with the actual dose-rate values for a specific type of radiation. Thus, we were able to compare the LTP dose rate with those for most of types of radiation, such as gamma rays[8,11-23], X-rays[24-29], ions[12,30-33], and protons[17,34,35], and we successfully estimated various deviation metrics for the above types of radiation (Table S4). However, in the case of electrons[9,36,37], UV rays[38,39], and alpha particles[8-11] only the absorbed doses used for DNA damage were reported rather than the dose-rate values. Therefore, we could not estimate any deviation metrics for these types of radiation.

Nevertheless, the calculated outcome from the comparative task indicated that the minimum estimated LTP dose rate of 0.21 Gy/s is the closest to the minimum dose rate of 0.07 Gy/s that was reported in the literature for protons even though it is three times higher (Table S4). In contrast, we obtained the larger deviation of a factor of 70 while comparing the minimum LTP dose rate with the minimum dose-rate value reported for X-rays.

Despite not having the dose-rate values for UV rays, electrons, and alpha particles, the dose rates obtained for LTP based on the dose-DNA damage correlations for these types of radiation showed values in the range of 1 Gy/s (Fig. S12), which is approximately five times higher than the LTP dose rate from modelling using the dose-DNA damage correlation for protons. Also, as seen in Eq. S8, for the minimum deviation of the LTP estimated dose rate to the literature value, a higher numerator value in this equation (LTP estimated dose rate) causes a larger deviation, which is the case for values obtained using dose-DNA correlation for electrons, UV rays, and alpha particles. In general, the absorbed doses used for DNA damage due to irradiation by UV rays, electrons, and alpha particles, had the same order of magnitude as the absorbed doses due to proton irradiation. Therefore, again, as seen in Eq. S8, if the dose-rate values (the denominator in this equation) are assumed to be of the same order as the dose rates for protons, alpha particles, electrons, and UV rays, the result gives a high deviation. Hence, our rough qualitative reasoning suggests that the dose-rate values for protons are the most comparable to the LTP dose rates.

Fricke dosimetry

We used a Fricke dosimeter that contains 1.4 mM Fe^{2+} (iron (II) sulfate heptahydrate from Sigma Aldrich Inc, St. Louis, MO) in 0.4 M H_2SO_4, and we saturated this ferrous sulfate solution with O_2.
We placed a cuvette with a ferrous sulfate solution (500 µl) and a small magnet (2.5 mm x 9 mm) on a magnetic stirrer, which was operated at a constant stirring rate. The solution surface was 2 cm from the tube orifice and had proper contact with a plasma jet. We irradiated solutions using one set of plasma process parameters: a voltage of 8 kV, a frequency of 1 kHz, and a gas flow rate of 2 slm, within several irradiation times between 0.5 and 5 min. After plasma irradiation, we collected 200 µl of the solution for spectrophotometric analysis. Control samples exposed to 3 min of a gas stream without plasma ignition showed no noticeable differences from untreated samples of ferrous sulfate.
We performed plasma irradiation and spectrophotometric readings at the temperature (T) of 24°C therefore we corrected the value of the extinction coefficient of the ferric ion using this reference temperature ($\varepsilon_{22°C}$ =2152 mol l^{-1} cm^{-1}) as previously shown (S9)[40].

$$\varepsilon_T = (1 + 0.007 \times (T - 22℃) \times \varepsilon_{22℃} \qquad \text{(S9)}$$

Then, we calculated an absorbed dose using the following Eq. S10:

$$D = \frac{\Delta OD \times N_A \times 100\ eV}{G(Fe^{3+}) \times t \times \varepsilon_T \times d \times \rho \times f \times 1000} \qquad \text{(S10)}$$

in which ΔOD corresponds to the change in the optical density of the irradiated solution relative to the non-irradiated solution at a wavelength of 304 nm, N_A is the Avogadro number (6.023×10^{23}), G (Fe^{3+}) is the chemical yield for the irradiation energy (15.6 ions/100eV), d is the optical pathway (0.7 cm), ρ is the solution density (1.024 kg/l), f is the conversion coefficient from eV to Gy (6.242×10^{15} [eV/g·Gy]). The data used to obtain a dose-rate value is presented in Fig. S13 and revealed a dose-rate value of 0.76 ± 0.02 Gy/s for a given set of plasma parameters. We noticed that the unstirred solution showed a 50 % lower dose rate.

Alanine dosimetry

We used alanine pellets (GEX corporation, Centennial, CO) with a 4 mm diameter and 2.35 mm thickness, and a composition of 93% of pure L-ɑ-alanine and 7% binder. We irradiated alanine pellets using the same process parameters as those for Fricke dosimetry, i.e., 8 kV, 1 kHz, and 2 slm, within a wide range of irradiation time (Fig. S14a). Similarly, as for the Fricke solution, the distance between the tube orifice and the pellet surface was 2 cm. After plasma irradiation, we inserted pellets into a custom-made quartz flat cell in an electron paramagnetic resonance (EPR) spectrometer cavity. We carried out EPR measurements using Bruker EMXplus spectrometer (Bruker Corporation, Billerica, MA) with ER4119HS standard resonator in X-band (9.77 GHz). We used the following EPR parameters: a magnetic field sweep width of 200 G, sweep time of 10 s, modulation amplitude of 1 G and frequency of 100 kHz, and microwave power of 2 mW with 50 average scans.
We determined the response of alanine dosimeters to plasma irradiation by comparing them to those after ^{60}Co gamma-ray irradiation. Alanine pellets were irradiated with gamma rays in the dose range of 5-40 Gy with Gammacell 220 Irradiator at the Notre Dame Radiation Laboratory. We measured the peak-to-peak amplitude of the central line of the EPR spectrum to calculate the absorbed dose (Fig. S14a). Fig. S14b compares the measured EPR spectrum of alanine pellets irradiated with ^{60}Co gamma rays and LTP. This comparison showed that the dose-rate value of alanine's response to plasma with respect to ^{60}Co equals 0.0101 ± 0.0003 Gy/s in the linear treatment range of 1-5 minutes for a given set of LTP process parameters.

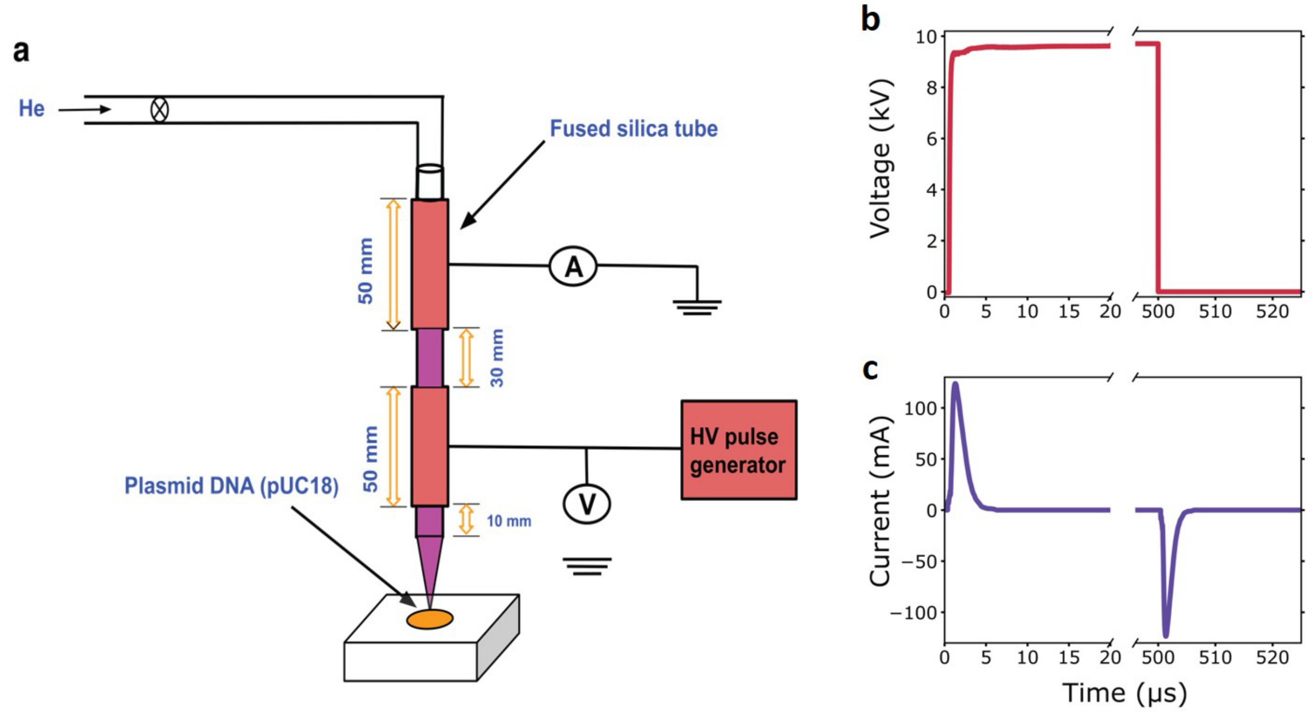

Fig. S1. Helium-fed, low-temperature plasma source. (a) Schematic diagram of the LTP source. Plasmid DNA in an aqueous solution was kept at a distance of 2 cm from the tube orifice. Tunable process parameters included the applied voltage, frequency, irradiation time, and feed gas flow rate. The process parameters were varied (Table S1) and incorporated into the DoE matrix. (b) Representative plot of the applied voltage and (c) current waveform for the plasma source with process parameters of 10 kV, 1 kHz, and 2 slm. The current was monitored through the grounded electrode, and the voltage was applied to the powered electrode.

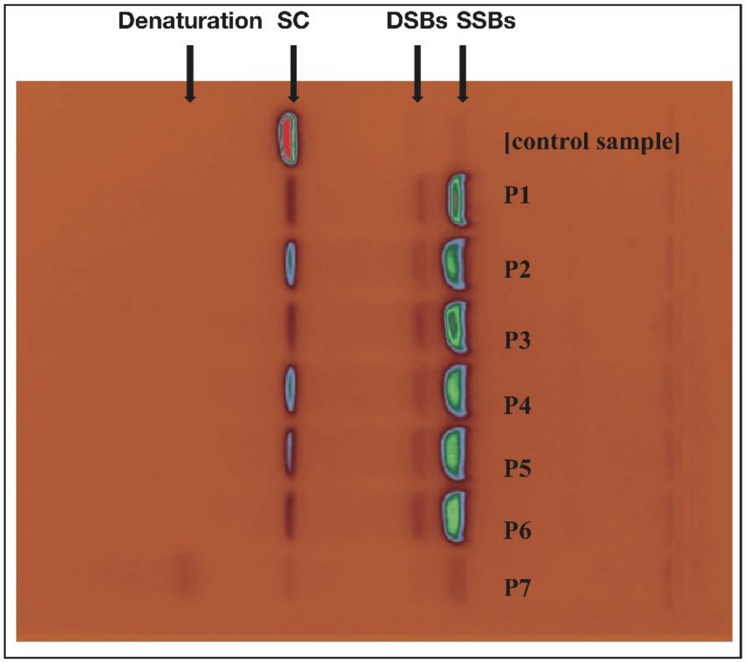

Fig. S2. UV fluorescence image of the electrophoresed gel. Representative image, not the actual data, showing four bands that correspond to the denaturation, undamaged supercoiled DNA (SC), single-strand breaks (SSBs), and double-strand breaks (DSBs). The bands in the topmost row correspond to the untreated (gas flow control) sample, and the bands in the other rows correspond to DNA irradiated by LTP at various combinations of process parameters. The P1 parameters are: 11 kV, 4 kHz, 10 s, and 2 slm; P2: 11 kV, 1 kHz, 20 s, and 2 slm; P3: 11 kV, 2 kHz, 20 s, and 2 slm; P4: 11 kV, 3 kHz, 20 s, and 2 slm; P5: 11 kV, 4 kHz, 20 s, and 2 slm; P6: 11 kV, 1 kHz, 30 s, and 2 slm, and P7: 11 kV, 2 kHz, 30 s, and 2 slm. The total DNA damage, including the sum of SSBs, DSBs, and denaturation, was computed from the relative fluorescence intensities.

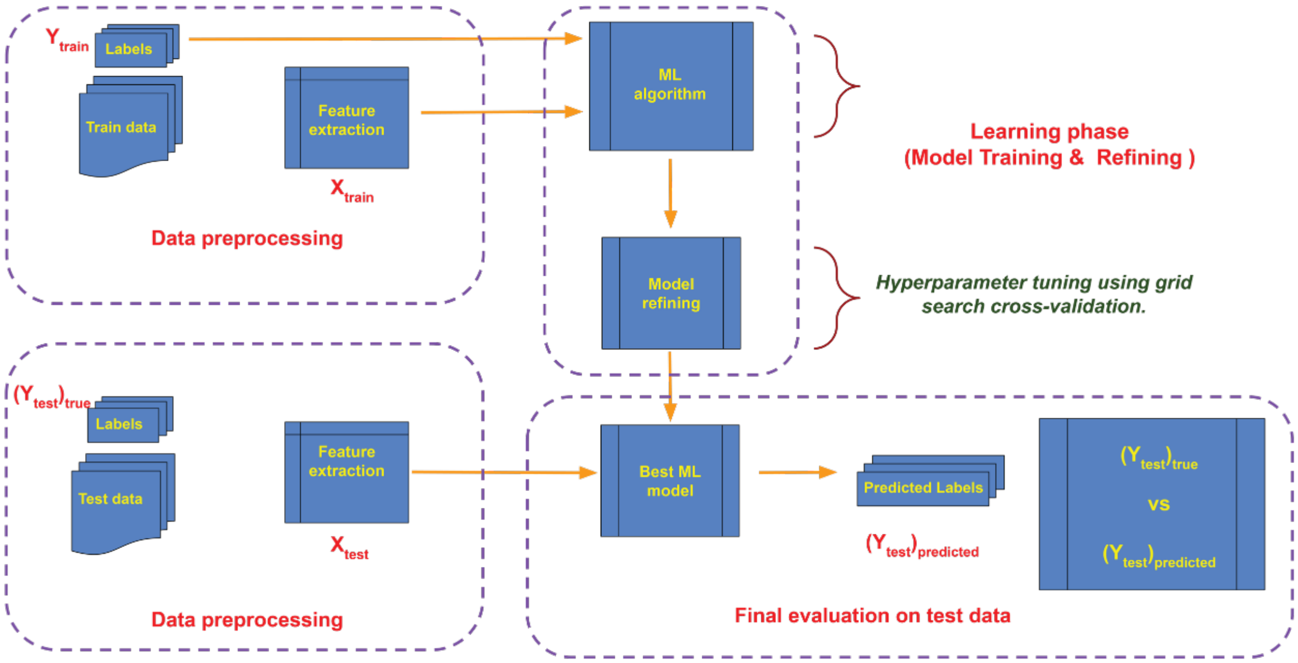

Fig. S3. Supervised machine learning workflow. Schematic diagram of the supervised machine learning workflow adopted for predictive modeling of total DNA damage. The features (X) correspond to the plasma process parameter vector: [voltage, frequency, irradiation time, flow rate], and labels (Y) correspond to the total DNA damage. The data from DoE were split into training/test data (75:25 ratio), and the training data were used for model learning and refining. Finally, the refined model after grid-search cross-validation was evaluated on the unaugmented test data values.

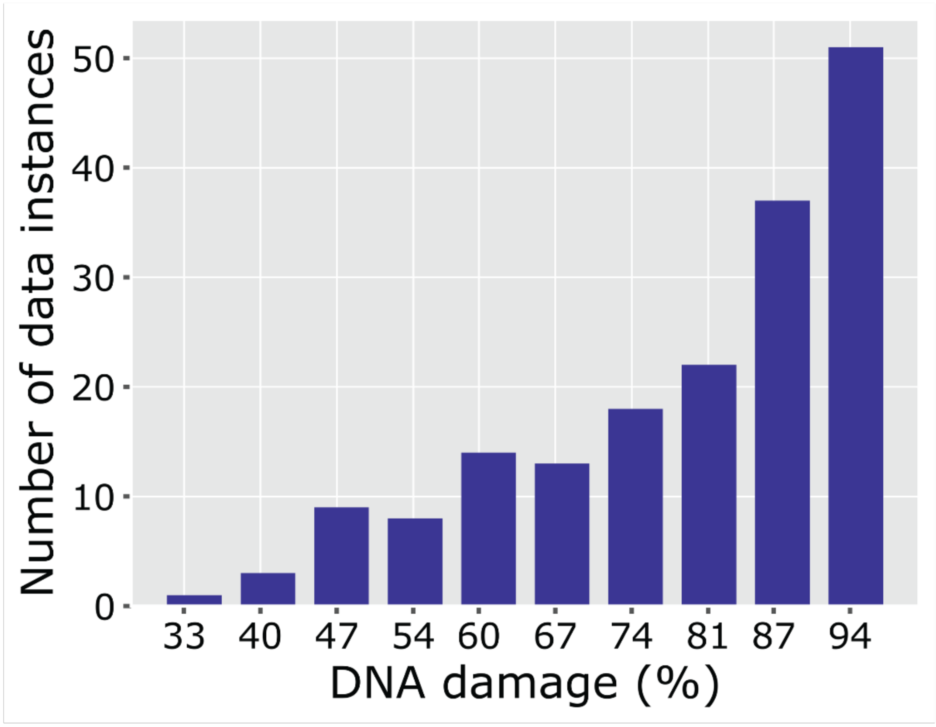

Fig. S4. Data distribution of the extent of plasma-induced DNA damage. Distribution of the total DNA damage indicated rare values for the low extents of DNA damage. These minority values corresponded to plasma-induced DNA damage at short irradiation times, mostly at 10 s.

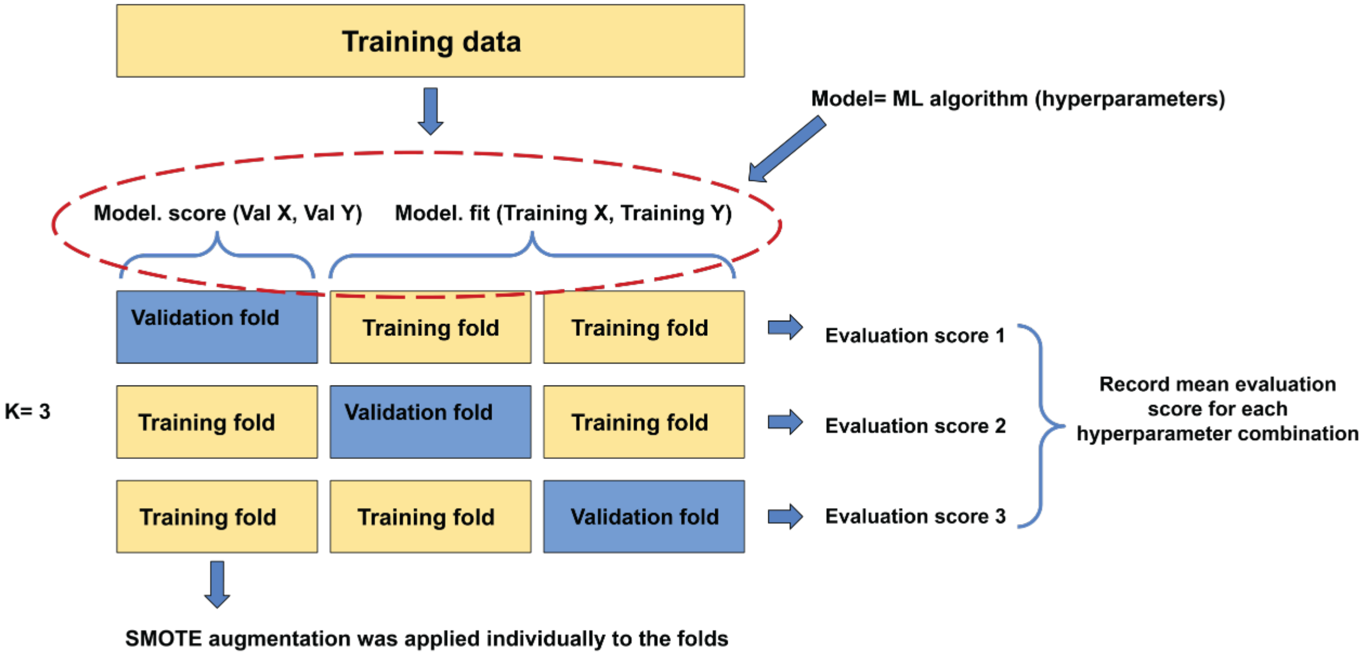

Fig. S5. Hyperparameter tuning with grid-search cross-validation. SMOTE augmentation was separately applied to the individual folds. The hyperparameter combination of the ML algorithm, which provided the best CV scores (threefold cross validation in this figure), was obtained through the grid-search process. The best hyperparameter acquired through the grid-search process was then used to evaluate the test data.

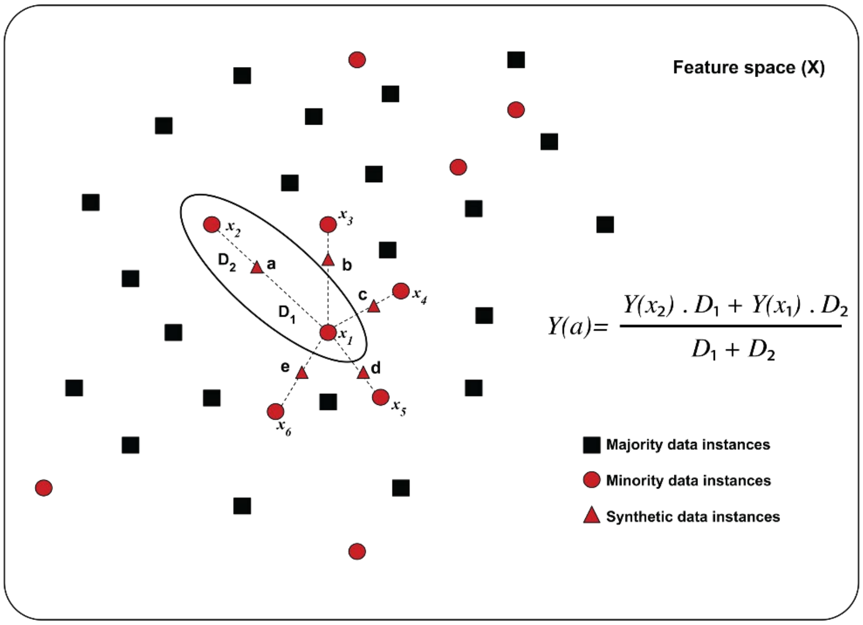

Fig. S6. Synthetic minority oversampling method (SMOTE). The minority data class corresponded to the extent of DNA damage that occurred at short irradiation times. The augmentation of each class of the data values involved generating synthetic data points between the nearest neighbors in the feature space (X). Inverse distance averaging was adopted to find the Y label of the generated data values.

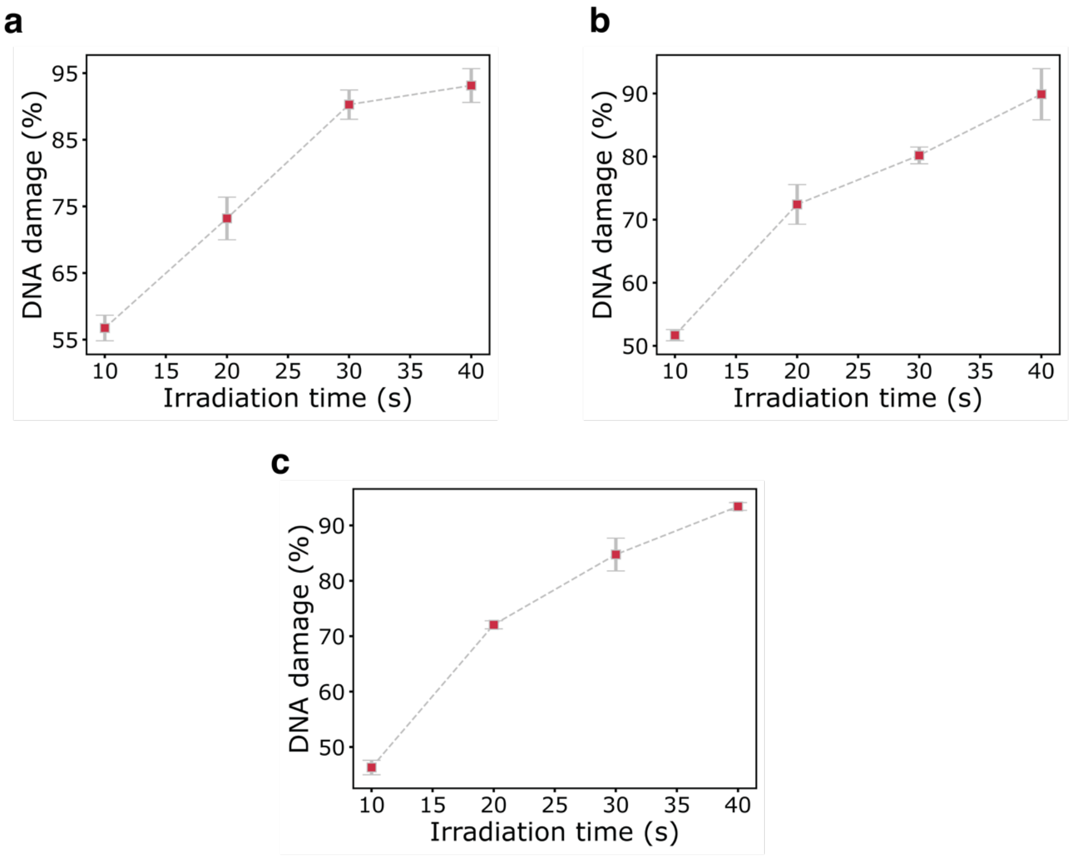

Fig. S7. Experimental data of time dependence for DNA damage. The extent of DNA damage is presented for three sets of process parameters: 10 kV, 2 kHz, and 2 slm (a), 8 kV, 2 kHz, and 4 slm (b), and 9 kV, 2 kHz, and 3 slm (c). This time dependence was used as the physical phenomenon applied to develop predictive models.

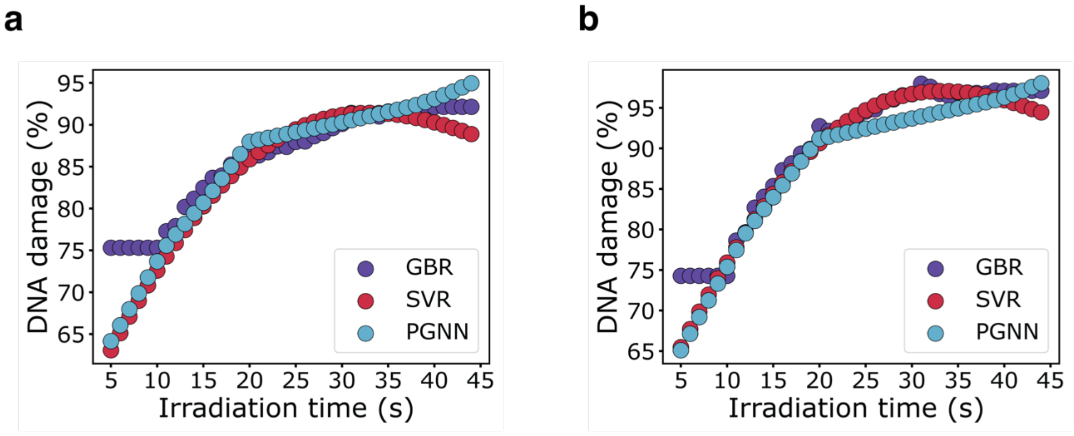

Fig. S8. Modeled data of time dependence for DNA damage. The extent of DNA damage is presented for two sets of process parameters: 11 kV, 3 kHz, and 2 slm (a), and 10 kV, 4 kHz, and 2 slm (b), using three ML algorithms: gradient boosting regressor (GBR), support vector regression (SVR), and physics-guided neural network (PGNN). Both models, i.e., GBR and SVR, provided the results that were physically inconsistent. GBR predicted a constant extent of DNA damage with the increase of time at short irradiation times. Whereas, SVR predicted a decrease in DNA damage with increased time at longer irradiation times. The results using PGNN provided the time dependence which obeyed the physical consistency.

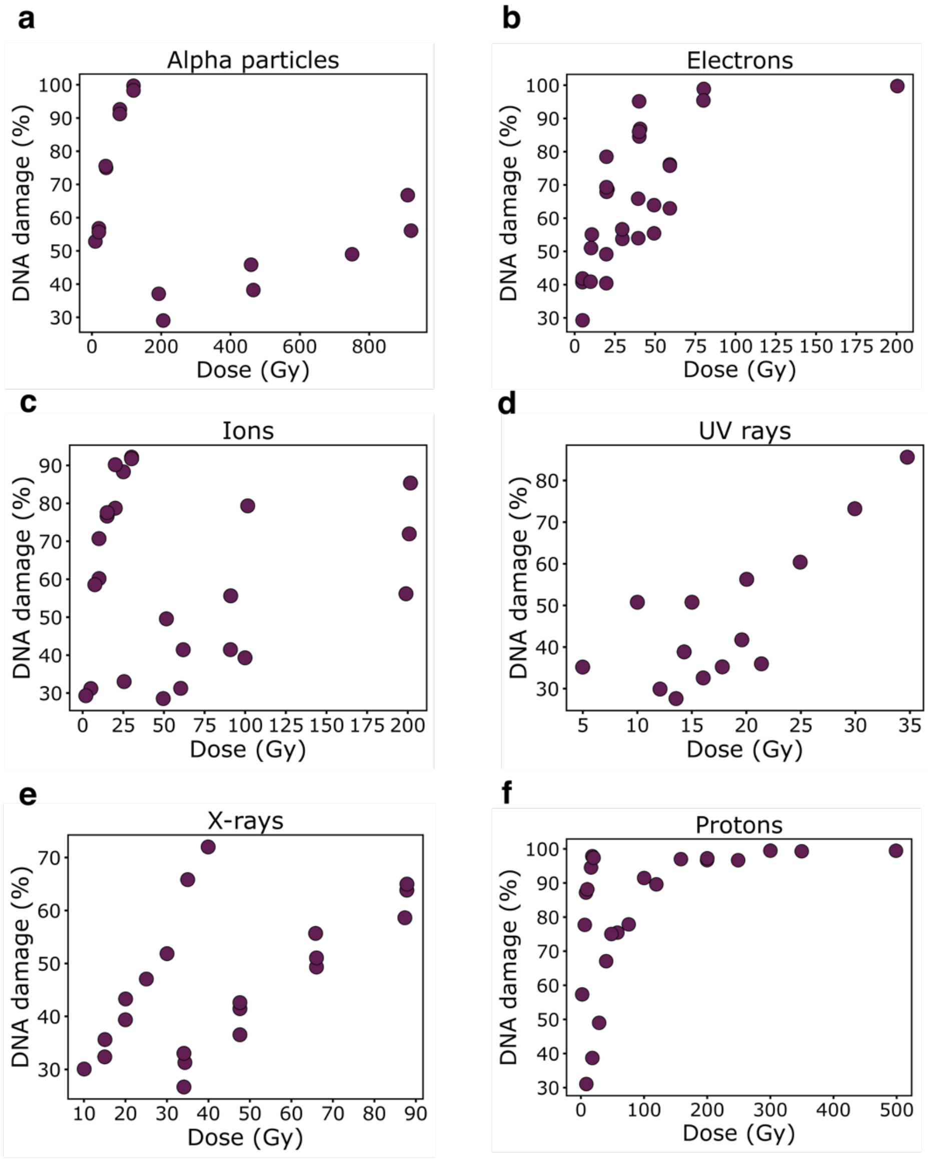

Fig. S9. Dose-DNA damage correlation for radiation sources. The plot of DNA damage vs. absorbed dose for alpha particles (a), electrons (b), ions (c), UV rays (d), X-rays (e), and protons (f).

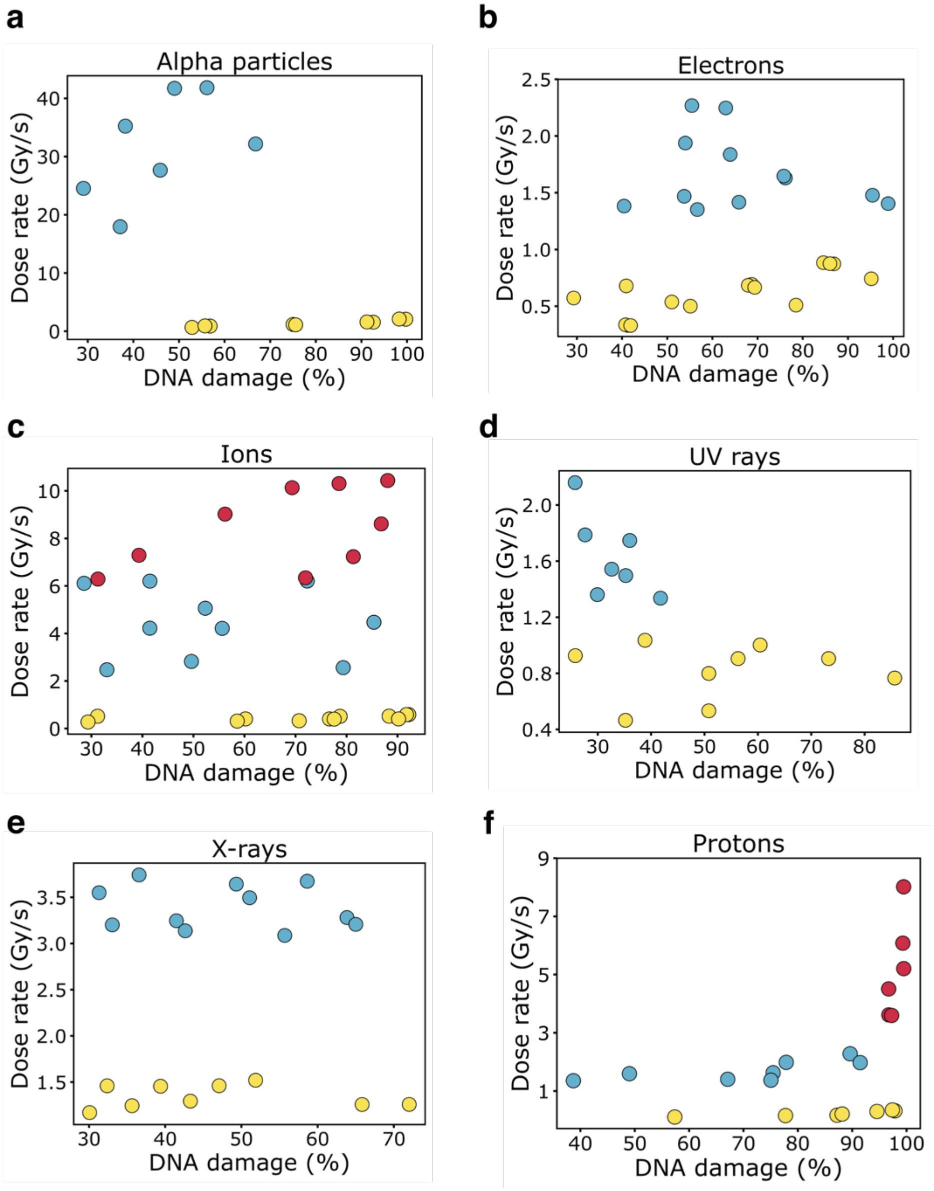

Fig. S10. Dose rate vs. DNA damage. Plots were obtained from modeling based on dose-DNA damage correlations for different types of radiation, i.e., alpha particles (a), electrons (b), ions (c), UV rays (d), X-rays (e), and protons (f). Data clustering indicated a formation of a few clusters for dose-rate values. Different colors represent clusters, and their values are listed in Table S3.

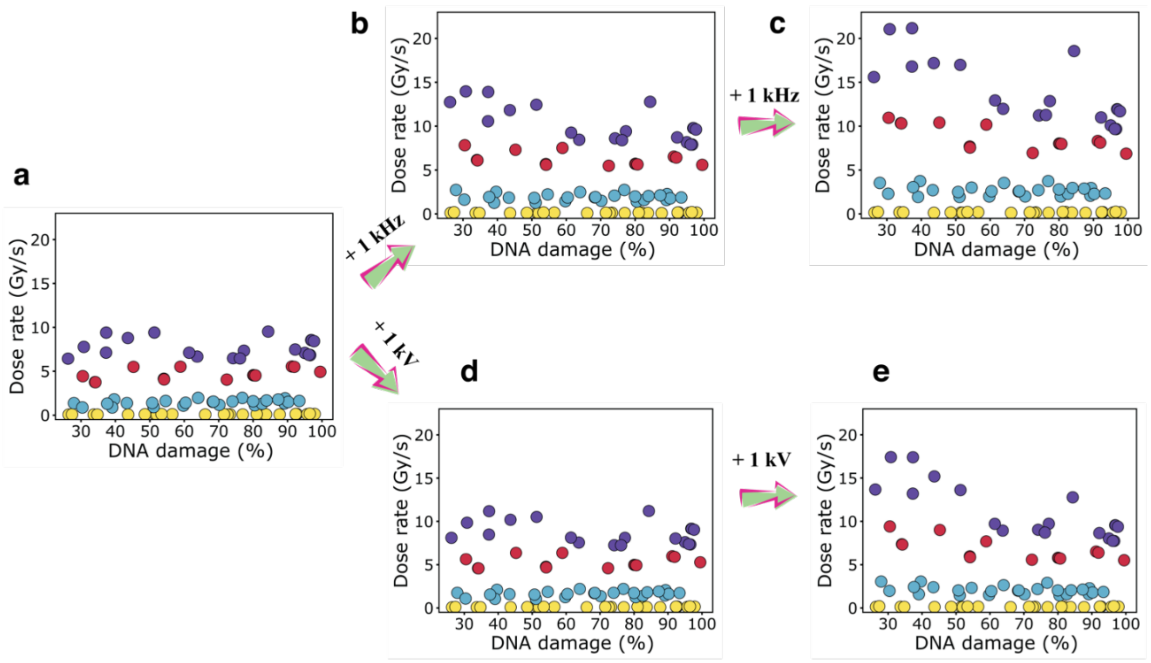

Fig. S11. Evolution of LTP dose-rate clusters with frequency and voltage. (a) The LTP dose-rate clusters as modeled using dose-DNA damage correlations for gamma-ray source for process parameters 8 kV, 0.5 kHz, and 2 slm. The evolution of LTP dose-rate clusters with increments of 1 kHz frequency when other process parameters were kept constant. Plasma process parameters: 8 kV, 1.5 kHz, and 2 slm (b), and 8 kV, 2.5 kHz, and 2 slm (c). The evolution of LTP dose rates with increments of 1 kV voltage when other process parameters were kept constant. Plasma process parameters: 9 kV, 0.5 kHz, and 2 slm (d), 10 kV, 0.5 kHz, and 2 slm (e).

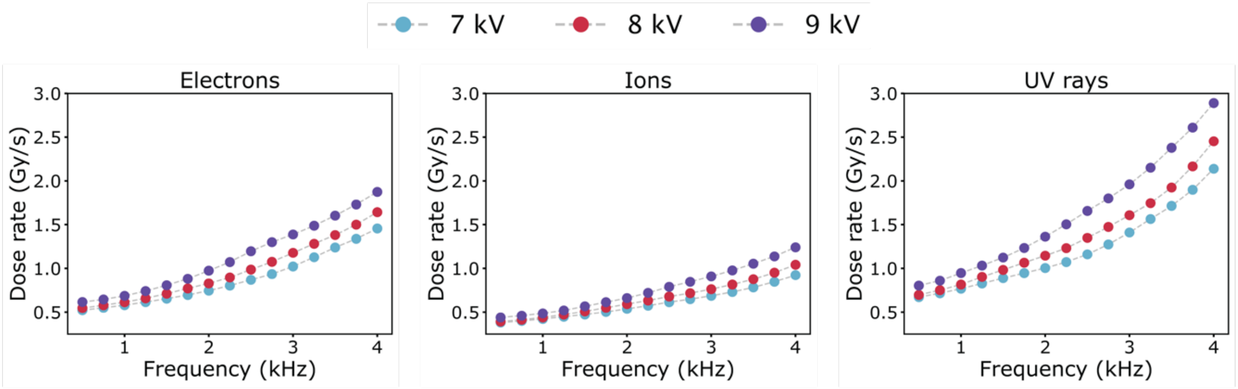

Fig. S12. Dose rate vs. frequency. Plots were obtained for average dose rates (a centroid of the lowest dose-rate cluster) from modeling based on dose-DNA damage correlations for different types of radiation, i.e., electrons (a), ions (b), and UV rays (c).

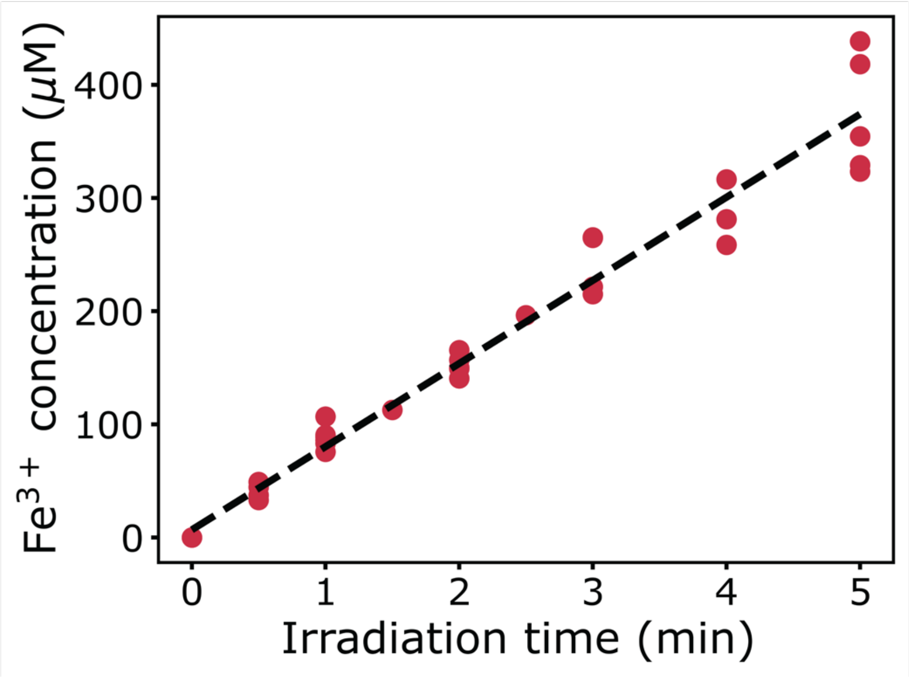

Fig. S13. Fricke dosimetry. The concentration of formed Fe^{3+} in the Fricke dosimeter solution vs. LTP irradiation time.

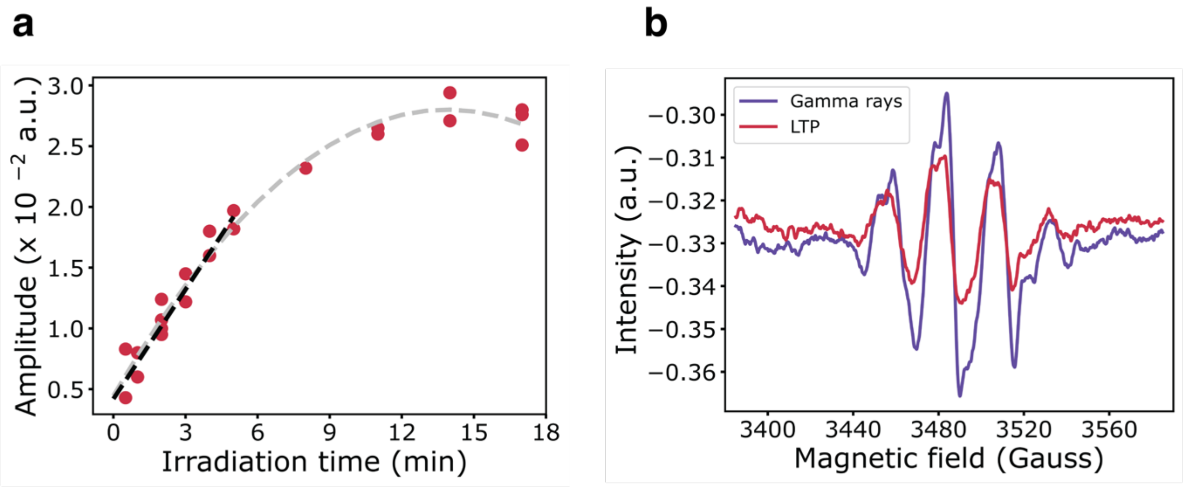

Fig. S14. Alanine dosimetry. (a) The signal amplitude of the central line of radicals formed in alanine pellets vs. LTP irradiation time. The signal shows a linear response within 5 min. (b) EPR spectra of radicals formed in alanine pellets after exposure to irradiation with gamma rays (10 Gy absorbed dose) and LTP (14 min irradiation time).

Table S1. Design of experiments (DoE) matrix. LTP process parameters that were varied experimentally.

Process parameter (unit)	Value
Voltage (kV)	8, 9, 10, 11
Frequency (kHz)	1, 2, 3, 4
Irradiation time (s)	10, 20, 30, 40
Flow rate (slm)	2, 3 , 4

Table S2. LTP dose-rate clusters obtained from the dose-DNA damage correlations for gamma rays. The mean (a centroid of the cluster) and the standard deviation of the dose rate for the respective clusters for LTP process parameters: 8 kV, 1 kHz, and 2 slm. Colors represent the corresponding data for the clusters plotted in Figs. 2f and 2g.

Cluster	Dose rate (Gy/s)
Cluster 1	0.11 $\pm$ 0.03
Cluster 2	1.63 $\pm$ 0.32
Cluster 3	5.28 $\pm$ 0.61
Cluster 4	8.72 $\pm$ 1.32

Table S3. LTP dose-rate clusters obtained from the dose-DNA damage correlations for different type of radiation. The mean (a centroid of the cluster) and the standard deviation of the dose rate for the respective clusters for LTP process parameters: 8 kV, 1 kHz, and 2 slm. Colors represent the corresponding data for the clusters plotted in Fig. S10.

Type of radiation	Cluster	Dose rate (Gy/s)
Alpha particles	Cluster 1	1.34 ± 0.48
	Cluster 2	31.59 ± 8.21
Electrons	Cluster 1	0.61 ± 0.18
	Cluster 2	1.67 ± 0.31
Ions	Cluster 1	0.44 ± 0.10
	Cluster 2	4.44 ± 1.40
	Cluster 3	8.41 ± 1.58
Protons	Cluster 1	0.23 ± 0.08
	Cluster 2	1.70 ± 0.32
	Cluster 3	5.17 ± 1.54
UV rays	Cluster 1	0.81 ± 0.19
	Cluster 2	1.63 ± 0.27
X-rays	Cluster 1	1.35 ± 0.12
	Cluster 2	3.39 ± 0.22

Table S4. Overview of the various metrics used in the comparative task. The statistical metrics include root-mean-squared error (RMSE), minimum RMSE, mean absolute error (MAE), and minimum MAE for the cluster with the lowest dose rate obtained for four types of radiation with the dose-rate value (Lit. Value) reported in the literature. The last column represents the ratio of the lowest dose rate estimated for LTP to the dose-rate value reported for a given type of radiation.

**Type of radiation**	**Protons**	**Gama rays**	**Ions**	**X-rays**
RMSE (Gy/s)	0.26	0.14	0.63	3.05
MAE (Gy/s)	0.24	0.14	0.62	3.00
Min RMSE (Gy/s)	0.16	0.01	0.37	1.07
Min MAE (Gy/s)	0.14	0.07	0.36	1.06
Lit. Value	0.07	0.01	0.03	0.02
RMSE / Lit. Value	3.71	13.9	25.20	152.50
MAE / Lit. Value	3.43	13.6	24.80	150.00
Min RMSE / Lit. Value	2.29	7.70	14.80	53.50
Min MAE / Lit. Value	2.00	7.30	14.40	53.00
Min LTP dose rate / Lit. value	3	8	15	70

Movie S1 (separate file). Centroid motion with changing frequency. The motion of the centroids for each cluster was obtained from the dose-DNA damage correlation for gamma rays modeled for frequencies from 0.5 to 4 kHz at the voltage of 8 kV.

Movie S2 (separate file). Centroid motion with changing voltage. The motion of the centroids for each cluster was obtained from the dose-DNA damage correlation for gamma rays modeled for voltages from 6 to 11 kV at the frequency of 1 kHz.

SI References

1. Adhikari, E. R., Samara,V. & Ptasinska, S. Influence of O_2 or H_2O in a plasma jet and its environment on plasma electrical and biochemical performances. J. Phys. D. Appl. Phys. 51 (2018). doi.org/10.1088/1361-6463/aab8f0
2. Han, X., Cantrell, W. A., Escobar, E. E. & S., Plasmid DNA damage induced by helium atmospheric pressure plasma jet. Eur. Phys. J. D. 68 (2014). doi.org/10.1140/epjd/e2014-40753-y
3. Adhikari, E. R. & Ptasinska, S. Correlation between helium atmospheric pressure plasma jet (APPJ) variables and plasma induced DNA damage. Eur. Phys. J. D. 70 (2016). doi.org/10.1140/epjd/e2016-70274-6
4. Daw, A., Karpatne, A., Watkins, W., Read, J. & Kumar, V. Physics-guided Neural Networks (PGNN): An Application in Lake Temperature Modeling. arXiv:1710.11431v3 [cs.LG] (21 September 2021)
5. Daw, A. et al. Physics-guided architecture (PGA) of neural networks for quantifying uncertainty in lake temperature modeling. arxiv.org/abs/1911.02682v1 [cs.LG] (6 November 2019)
6. Read, J. S. et al. Process-Guided Deep Learning Predictions of Lake Water Temperature. Water Resour. Res. 55, 9173–9190 (2019). doi.org/10.1029/2019WR024922
7. Muralidhar, N. et al. Phynet: Physics guided neural networks for particle drag force prediction in assembly. Proc. 2020 SIAM Int. Conf. Data Mining, SDM 2020, 559–567 (2020). doi.org/10.1137/1.9781611976236.63
8. Prise, K. M., Pullar, C. H. L. & Michael, B. D. A study of endonuclease III-sensitive sites in irradiated DNA: detection of α-particle-induced oxidative damage. Carcinogenesis. 20, 905-9 (1999). doi.org/10.1093/carcin/20.5.905
9. Reissig, F. et al. The effect of hypoxia on the induction of strand breaks in plasmid DNA by alpha-, beta-and Auger electron-emitters ^{223}Ra, ^{188}Re, 99mTc and DNA-binding 99mTc-labeled pyrene. Nucl. Med. Biol. 80, 65–70 (2020). doi.org/10.1016/j.nucmedbio.2020.01.003
10. Yokoya, A., Cunniffe, S. M. T., Stevens, D. L. & O'Neill, P. Effects of hydration on the induction of strand breaks, base lesions, and clustered damage in DNA films by α-radiation. J. Phys. Chem. B. 107, 832–837 (2003). doi.org/10.1021/jp0270708
11. Hodgkins, P. S., O'Neill, P., Stevens, D. & Fairman, M. P. The severity of alpha-particle-induced DNA damage is revealed by exposure to cell-free extracts. Radiat. Res. 146, 660–667 (1996). doi.org/10.2307/3579382
12. Terato, H. et al. Quantitative analysis of isolated and clustered DNA damage induced by gamma-rays, carbon ion beams, and iron ion beams. J. Radiat. Res. 49, 133–146 (2008). doi.org/10.1269/jrr.07089
13. Kouass Sahbani, S., Rezaee, M., Cloutier, P., Sanche, L. & Hunting, D. J. Non-DSB clustered DNA lesions induced by ionizing radiation are largely responsible for the loss of plasmid DNA functionality in the presence of cisplatin. Chem. Biol. Interact. 217, 9–18 (2014). doi.org/10.1016/j.cbi.2014.04.004
14. Gulston, M., Fulford, J., Jenner, T., de Lara, C. & O'Neill, P. Clustered DNA damage induced by γ radiation in human fibroblasts (HF19), hamster (V79-4) cells and plasmid DNA is revealed as Fpg and Nth sensitive sites. Nucleic Acids Res. 30, 3464–3472 (2002). doi.org/10.1093%2Fnar%2Fgkf467
15. Milligan, J. R., Aguilera, J. A., Paglinawan, R. A., Ward, J. F. & Limoli, C. L. DNA strand break yields after post-high LET irradiation incubation with endonuclease-III and evidence for hydroxyl radical clustering. Int. J. Radiat. Biol. 77, 155–164 (2001). doi.org/10.1080/09553000010013445
16. Milligan, J. R., Aguilera, J. A., Nguyen, T.-T. D., Paglinawan, R. A. & Ward, J. F. DNA strand-break yields after post-irradiation incubation with base excision repair endonucleases implicate hydroxyl radical pairs in double-strand break formation. Int. J. Radiat. Biol. 76, 1475–1483 (2000). doi.org/10.1080/09553000050176234
17. Sui, L. et al. Clustered DNA damage induced by protons radiation in plasmid DNA . Chinese Sci. Bull. 58, 3217–3223 (2013). doi.org/10.1007/s11434-013-5940-x
18. Yokoya, A., Cunniffe, S. M. T. & O'Neill, P. Effect of hydration on the induction of strand breaks and base lesions in plasmid DNA films by γ-radiation. J. Am. Chem. Soc. 124, 8859–8866 (2002). doi.org/10.1021/ja025744m
19. Peak, J. G., Ito, T., Robb, F. T. & Peak, M. J. DNA damage produced by exposure of supercoiled plasmid DNA to high-and low-LET ionizing radiation: effects of hydroxyl radical quenchers. Int. J. Radiat. Biol. 67, 1–6 (1995). doi.org/10.1080/09553009514550011

20. Sahbani, S. K., Girouard, S., Cloutier, P., Sanche, L. & Hunting, D. J. The relative contributions of DNA strand breaks, base damage and clustered lesions to the loss of DNA functionality induced by ionizing radiation. Radiat. Res. 181, 99–110 (2014). doi.org/10.1667/rr13450.1
21. Milligan, J. R., Aguilera, J. A. & Ward, J. F. Variation of single-strand break yield with scavenger concentration for plasmid DNA irradiated in aqueous solution. Radiat. Res. 133, 151–157 (1993). doi.org/10.2307/3578350
22. Milligan, J. R. et al. Methylperoxyl radicals as intermediates in the damage to DNA irradiated in aqueous dimethyl sulfoxide with gamma rays. Radiat. Res. 146, 436–443 (1996). doi.org/10.2307/3579305
23. Hodgkins, P. S., Fairman, M. P. & O'Neill, P. Rejoining of gamma-radiation-induced single-strand breaks in plasmid DNA by human cell extracts: dependence on the concentration of the hydroxyl radical scavenger, Tris, Radiat. Res. 145, 24–30 (1996). doi.org/10.2307/3579191
24. Agrawala, P. K. et al. Induction and repairability of DNA damage caused by ultrasoft X-rays: Role of core events. Int. J. Radiat. Biol. 84, 1093–1103 (2008). doi.org/10.1080/09553000802478083
25. Morozov, K. V. et al. Radiosensitization by gold nanoparticles: impact of the size, dose rate, and photon energy. Nanomaterials. 10, 952 (2020). doi.org/10.3390/nano10050952
26. Brezeanu, M., Träger, F. & Hubenthal, F. Scanning force microscopy studies of X-ray-induced double-strand breaks in plasmid DNA. J. Biol. Phys. 35, 163 (2009). doi.org/10.1007/s10867-009-9137-0
27. Fujii, K., Yokoya, A. & Shikazono, N. Induction of single strand breaks, and base lesions in plasmid DNA films induced by carbon, nitrogen, and oxygen KLL Auger process. Int. J. Radiat. Biol. 84, 1104–1111 (2008). doi.org/10.1080/09553000802482564
28. Souici, M. et al. DNA strand break dependence on Tris and arginine scavenger concentrations under ultra-soft X-ray irradiation: the contribution of secondary arginine radicals. Radiat. Environ. Biophys. 55, 215–228 (2016). doi.org/10.1007/s00411-016-0642-9
29. Boichot, S. et al. Investigation of radiation damage in DNA by using atomic force microscopy. Radiat. Prot. Dosimetry. 99, 143–146 (2002). doi.org/10.1093/oxfordjournals.rpd.a006745
30. Shiina, T. et al. Induction of DNA damage, including abasic sites, in plasmid DNA by carbon ion and X-ray irradiation. Radiat. Environ. Biophys. 52, 99–112 (2013). doi.org/10.1007/s00411-012-0447-4
31. Urushibara, A. et al. LET dependence of the yield of single-, double-strand breaks and base lesions in fully hydrated plasmid DNA films by $^4He^{2+}$ ion irradiation. Int. J. Radiat. Biol. 84, 23–33 (2008). doi.org/10.1080/09553000701616072
32. Milligan, J. R., Aguilera, J. A., Paglinawan, R. A., Ward, J. F. & Limoli, C. L. DNA strand break yields after post-high LET irradiation incubation with endonuclease-III and evidence for hydroxyl radical clustering. Int. J. Radiat. Biol. 77, 155–164 (2001). doi.org/10.1080/09553000010013445
33. Yogo, K. et al. Potential mechanisms for protective effect of D-methionine on plasmid DNA damage induced by therapeutic carbon ions. Radiat. Res. 193, 513–519 (2020). doi.org/10.1667/rr15502.1
34. Vyšín, L. et al. Proton-induced direct and indirect damage of plasmid DNA. Radiat. Environ. Biophys. 54, 343–352 (2015). doi.org/10.1007/s00411-015-0605-6
35. Leloup, C. et al. Evaluation of lesion clustering in irradiated plasmid DNA. Int. J. Radiat. Biol. 81, 41–54 (2005). doi.org/10.1080/09553000400017895
36. Folkard, M. et al. Measurement of DNA damage by electrons with energies between 25 and 4000 eV. Int. J. Radiat. Biol. 64, 651–658 (1993). doi.org/10.1080/09553009314551891
37. Boudaïffa, B., Hunting, D., Cloutier, P., Huels, M. A. & Sanche, L. Induction of single-and double-strand breaks in plasmid DNA by 100–1500 eV electrons. Int. J. Radiat. Biol. 76, 1209–1221 (2000). doi.org/10.1080/09553000050134447
38. Śmiałek, M. A., Moore, S. A., Mason N. J. & Shuker, D. E. G. Quantification of radiation-induced single-strand breaks in plasmid DNA using a TUNEL/ELISA-based assay. Radiat. Res. 172, 529–536 (2009). doi.org/10.1667/rr1684.1
39. Śmialek, M. A. et al. VUV irradiation studies of plasmid DNA in aqueous solution, J. Phys.: Conf. Ser. 101, 012020. doi.org/10.1088/1742-6596/101/1/012020
40. Spinks, J. W. T. & Woods, R. J. An introduction to Radiation Chemistry, John-Wiley and Sons, 2nd ed., New York, p. 95, (1976)
